# Nonconditioned ADA-SCID gene therapy reveals ADA requirement in the hematopoietic system and clonal dominance of vector-marked clones

**DOI:** 10.1016/j.omtm.2021.10.003

**Published:** 2021-10-16

**Authors:** Toru Uchiyama, Sirirat Takahashi, Kazuhiko Nakabayashi, Kohji Okamura, Kaori Edasawa, Masafumi Yamada, Nobuyuki Watanabe, Emi Mochizuki, Toru Yasuda, Akane Miura, Motohiro Kato, Daisuke Tomizawa, Makoto Otsu, Tadashi Ariga, Masafumi Onodera

**Affiliations:** 1 Department of Human Genetics, National Center for Child Health and Development, Tokyo, Japan; 2 Department of Maternal-Fetal Biology, National Center for Child Health and Development, Tokyo, Japan; 3 Department of System Biomedicine, National Center for Child Health and Development, Tokyo, Japan; 4 Department of Pediatrics, Faculty of Medicine and Graduate School of Medicine, Hokkaido University, Sapporo, Japan; 5 Department of Pediatric Hematology and Oncology Research, National Center for Child Health and Development, Tokyo, Japan; 6 Children’s Cancer Center, National Center for Child Health and Development, Tokyo, Japan; 7 Department of Transfusion and Cell Transplantation, Kitasato University School of Medicine, Kanagawa, Japan

**Keywords:** ADA-SCID, retroviral vector, nonconditioned gene therapy, clonal dominance, ADA activity, insertional mutagenesis, bone marrow microenvironment

## Abstract

Two patients with adenosine deaminase (ADA)-deficient severe combined immunodeficiency (ADA-SCID) received stem cell-based gene therapy (SCGT) using GCsapM-ADA retroviral vectors without preconditioning in 2003 and 2004. The first patient (Pt1) was treated at 4.7 years old, and the second patient (Pt2), who had previously received T cell gene therapy (TCGT), was treated at 13 years old. More than 10 years after SCGT, T cells showed a higher vector copy number (VCN) than other lineages. Moreover, the VCN increased with differentiation toward memory T and B cells. The distribution of vector-marked cells reflected variable levels of ADA requirements in hematopoietic subpopulations. Although neither patient developed leukemia, clonal expansion of SCGT-derived clones was observed in both patients. The use of retroviral vectors yielded clonal dominance of vector-marked clones, irrespective of the lack of leukemic changes. Vector integration sites common to all hematopoietic lineages suggested the engraftment of gene-marked progenitors in Pt1, who showed severe osteoblast (OB) insufficiency compared to Pt2, which might cause a reduction in the stem/progenitor cells in the bone marrow (BM). The impaired BM microenvironment due to metabolic abnormalities may create space for the engraftment of vector-marked cells in ADA-SCID, despite the lack of preconditioning.

## Graphical abstract

**Figure:**
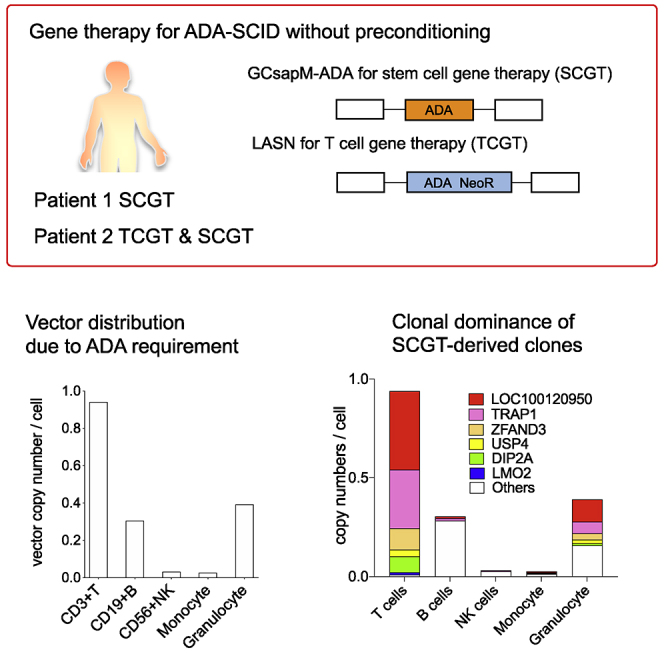

## Teaser

The distribution of vector-marked cells reflected variable levels of ADA requirements in hematopoietic subpopulations in ADA-SCID gene therapy. The use of retroviral vectors yielded clonal dominance of vector-marked clones, irrespective of the lack of leukemic changes.

## Introduction

Defects in adenosine deaminase (ADA), a crucial enzyme in the purine salvage pathway, result in autosomal recessive severe combined immunodeficiency (SCID).^1^^,^^2^ Stem cell-based gene therapy (SCGT) has been developed as a treatment for patients with primary immunodeficiencies who lack suitable donors for hematopoietic stem cell (HSC) transplantation.^3^^,^^4^ In SCGT trials for ADA-deficient SCID (ADA-SCID) patients, multi-lineage engraftment of transduced cells has been achieved by administrating busulfan before infusion, which creates space for the engraftment of manipulated HSCs in the bone marrow (BM). A high degree of immune reconstitution has been observed in treated patients and enabled them to discontinue enzyme replacement therapy (ERT) and immunoglobulin (Ig) replacement.^5^, ^6^, ^7^, ^8^, ^9^, ^10^ Two Japanese patients with ADA-SCID were treated with SCGT in 2003 and 2004 without cytoreductive conditioning. Partial and temporal reconstitution of the immune system was observed.^11^^,^^12^ In this study, we analyzed the peripheral blood (PB) and BM of these patients for long-term engraftment of vector-marked cells. The vector distributions reflected the extent of the ADA requirements in hematopoietic subpopulations. Therefore, transplantation without preconditioning chemotherapy may also be effective for vector insertions and provide an adequate BM microenvironment for the long-term engraftment of vector-marked cells in ADA-SCID gene therapy.

## Results

### Patients and clinical trial protocol

The characteristics of the patients and detailed information about the clinical trials have been reported previously.^11^^,^^13^ Briefly, the first patient (Pt1) was a female and developed clinical symptoms 15 days after birth, and ERT using polyethylene glycol-modified ADA (PEG-ADA) was commenced. SCGT using GCsapM-ADA retroviral vectors was performed at the age of 4.7 years. PEG-ADA was withdrawn, and no cytoreductive therapy was administered before SCGT. The second patient (Pt2) was a male and showed delayed onset as he was affected with severe pneumonia 8 months after birth and started PEG-ADA at 1.5 years old. He received T cell gene therapy (TCGT) with LASN retroviral vectors at 4.5 years old, and insufficient immune reconstitution resulted in the necessity for SCGT at 13.0 years old. TCGT consisted of repeated infusions of autologous gene-modified T cells with continuous ERT, and SCGT was performed under the same protocol as in Pt1. After more than 10 years, both patients showed partial immune reconstitution. Pt1 suffered from mild viral and bacterial infections in the years following the treatment, and her lymphocyte count remained at 200–300/μL. Pt2 showed a relatively higher lymphocyte count (300–1,000/μL) with a response to mitogen. However, he occasionally had mild viral infections, including skin lesions due to verruca vulgaris. Both patients required Ig supplementation to maintain serum IgG levels over 800 mg/dL (Figure S1).

### Engraftment of gene-corrected cells in the hematopoietic system

**Figure 1:**
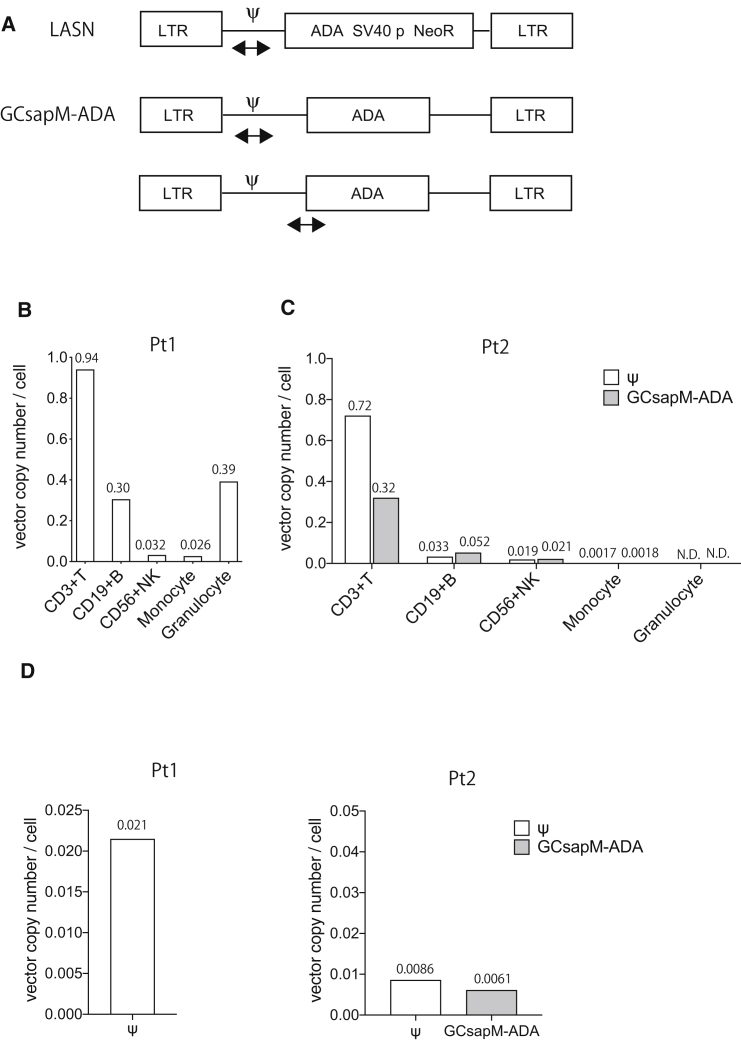
Gene marking of retroviral vectors after gene therapy (A) Target sequences of retroviral vectors for calculating vector copy number (VCN) using droplet digital PCR (ddPCR). The sequence of the packaging signal (Ψ) was common between the GCsapM-ADA and LASN retroviral vectors. For Pt2, the VCN was also determined using primers and probes against the GCsapM-ADA-specific sequence. (B and C) The VCN in sorted cell lineages of the peripheral blood from the patients. In Pt2, the VCN was calculated based on the Ψ and GCsapM-ADA sequences. (D) The VCN in the bone marrow (BM). CD34^+^ cells were isolated from the BM cells and were then analyzed. SV40 p, SV40 promoter; NeoR, neomycin-resistant gene; N.D., not detected.

We calculated the vector copy number (VCN) in the hematopoietic subpopulations to investigate the long-term engraftment of gene-corrected cells. PB cells were sorted into CD3^+^ T cells, CD19^+^ B cells, CD56^+^ natural killer (NK) cells, CD14^+^ monocytes, and CD15^+^ granulocytes, and then genomic DNA was extracted. The VCN was calculated using droplet digital PCR (ddPCR) with primers and probe against the packaging signal (Ψ; Figure 1A). In Pt1, the VCN in T cells was approximately 0.94 per cell, whereas other cell lineages, including B cells, NK cells, monocytes, and granulocytes, showed a VCN of 0.026–0.39 (Figure 1B). Consistent with previous reports, the exogenous expression of ADA provided a definitive selective advantage to the T cell lineage but not to other lineages. Although Pt1 only received SCGT, Pt2 received TCGT followed by SCGT, and, therefore, we also determined the VCN by tracking the GCsapM-ADA-specific sequence to examine the engraftment of SCGT-derived cells in Pt2 (Figure 1A). Pt2 showed a lower VCN (0.32) of GCsapM-ADA in T cells as compared to Pt1. However, as reported elsewhere (unpublished data), the remainder of the T cells contained the LASN vector used in TCGT, and, therefore, the total VCN calculated on the packaging signals was around 0.7 (Figure 1C). A small population of monocytes showed the integration of GCsapM-ADA, but granulocytes showed no integration of the vector. The lower frequency of GCsapM-ADA in T cells may be a consequence of inhibited T cell differentiation from the SCGT-derived HSC/hematopoietic progenitor cells (HPCs) by the presence of TCGT-derived T cells. However, the small number of integrations into myeloid lineages implied the loss of the common progenitors in Pt2. The BM CD34-positive cells from Pt1 showed a higher VCN (0.021) than that of Pt2 (Figure 1D). Detection of vector-marked cells in all hematopoietic lineages and a relatively higher VCN in the BM imply that gene-marked HSC/HPC subsets engrafted in Pt1 despite the lack of preconditioning.

### Vector distributions in subdivided subsets of T and B cells

**Figure 2:**
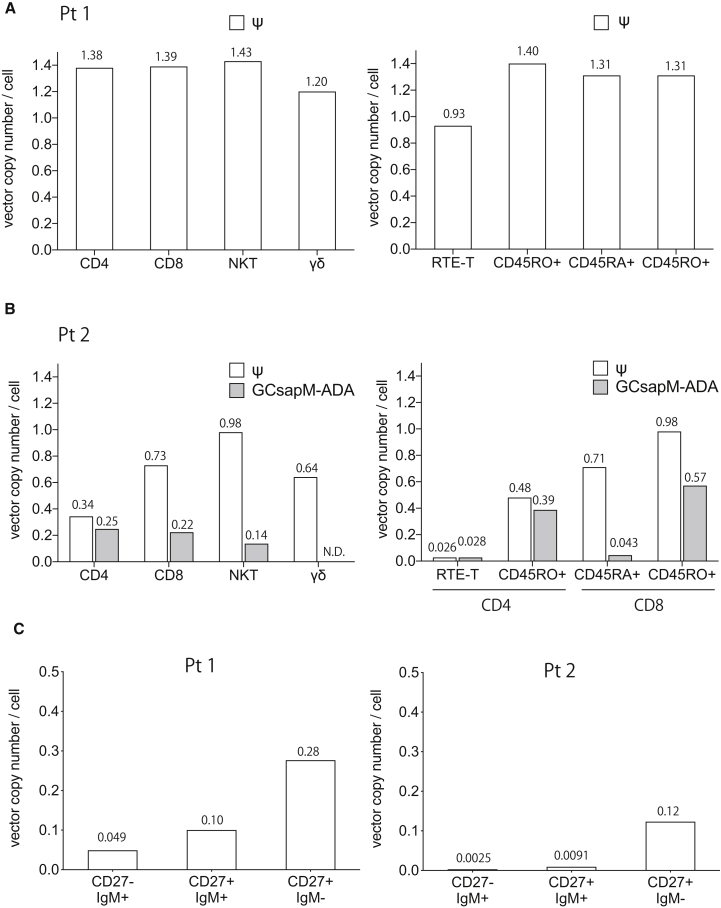
Vector distribution in subdivided subsets of T and B cells (A) The VCN in T cell subpopulations in Pt1. Sorted subsets including CD4^+^, CD8^+^, NKT, and γδ T cells were analyzed for the presence of the Ψ sequence. The VCN was also determined in subpopulations of differentiation stages, such as RTE-T (CD4^+^CD45RA^+^CD31^+^), memory CD4 (CD4^+^CD45RA), naive CD8 (CD8^+^CD45RA^+^), and memory CD8 (CD8^+^CD45RO^+^) cells. (B) The VCN in T cell subpopulations in Pt2. ddPCR analysis of Ψ and GCsapM-ADA-specific sequences was performed. (C) The VCN of Ψ in subsets of B cells, including naive (CD27^−^IgM^+^), IgM memory (CD27^+^IgM^+^), and class-switched memory (CD27^+^IgM^−^) B cells.

We further investigated the distribution of the retroviral vectors at differentiation stages of T and B cells. CD3^+^ T cells from both patients were first sorted into CD4^+^ T, CD8^+^ T, CD3^+^CD56^+^ NKT, and γδ T cells. Among the CD4^+^ and CD8^+^ T cells, recent thymic emigrant T (RTE-T: CD4^+^CD45RA^+^CD31^+^), memory CD4 (CD4^+^CD45RO^+^), naive CD8 (CD8^+^CD45RA^+^), and memory CD8 (CD8^+^CD45RO^+^) cells were separated and analyzed for vector integration. In Pt1, most of the T cell subpopulations showed a VCN > 1 (Figure 2A). RTE-T cells, which are a very early stage of naive CD4^+^ T cells, showed a slightly lower VCN (0.93). In Pt2, the frequency of GCsapM-ADA increased along with differentiation from naive to memory cells (unpublished data), causing changes in the total VCN (Figure 2B). In the RTE-T and naive CD8^+^ T subsets, T cells without vector integration were present, implying that these subsets have a lower requirement for ADA. Memory T cells required a high level of ADA supplied by the GCsapM-ADA vector rather than the LASN vector.

We also fractionated CD19^+^ B cells into CD27^−^IgM^+^ B cells (naive B cells), CD27^+^IgM^+^ B cells (IgM memory), and CD27^+^IgM^−^ B cells (class-switched memory) (Figure 2C). In Pt1, whereas naive B cells showed a much lower value (0.049) than T cells, an increase in total VCN was observed along with differentiation toward class-switched memory cells. Remarkably, in Pt2, vector integration was barely detectable (VCN = 0.0025) in naive B cells; however, class-switched memory B cells showed a significantly increased VCN (0.12). Whereas the presence of ADA did not show any selective advantage in naive B cells, the differentiation and maturation processes required a higher level of ADA activity. Vector distribution in the subdivided populations of T and B cells indicated an increase in ADA activity along with differentiation from naive to memory cells.

### Analysis of vector integration sites (ISs) in the patients

**Figure 3:**
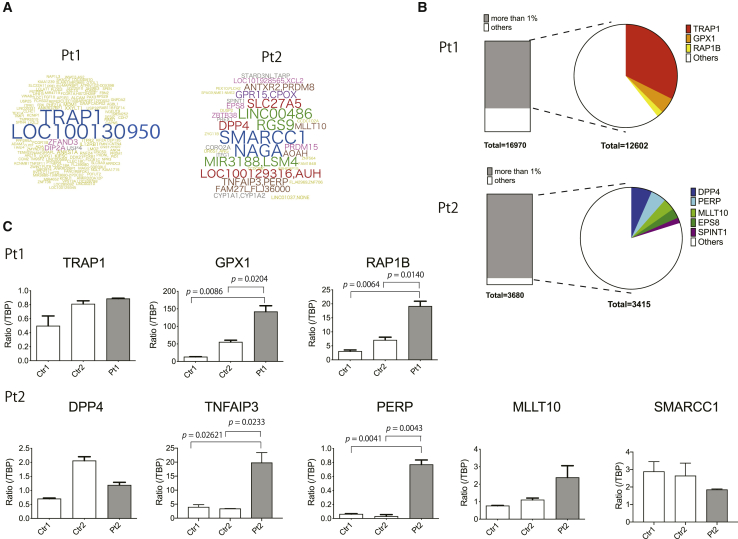
Vector integration site (IS) analysis (A) The frequency of targeted genes with vector integration 13 years after SCGT. The size corresponds to the frequency of the gene into or near the location where the retroviral vector was integrated. (B) ISs into/near annotated cancer-related genes with frequencies higher than 1% of the total reads. (C) Transcription levels of cancer-related genes with vector integration in Pt1 and Pt2. The top three integrations were analyzed for their impact on the expressions of genes near the ISs. One integration in Pt2 was located between *TNFAIP3* and *PERP*, and the expression of both genes was analyzed. Since the clone with GCsapM-ADA integration into the *SMARCC1* locus clonally expanded, we also analyzed the transcription of *SMARCC1* in Pt2.

Neither patient received preconditioning, which exposed the transduced cells to proliferative stress. Therefore, we explored the vector ISs to reveal whether genetic factors related to vector insertion into chromosomes might cause prolonged survival of the specific clones in both patients. We established a capture system targeting the sequence of the vector long terminal repeat (LTR), followed by high-throughput sequencing using next-generation sequencing (NGS). A total of 417 ISs with clonal dominance of specific integrations (*LOC100130950* and tumor necrosis factor [TNF] receptor-associated protein 1 [*TRAP1*]) were detected (Figure 3A; Table S1) in Pt1. Vector ISs in Pt2 have been reported elsewhere (unpublished data), and most of the highly frequent ISs except *SMARCC1* were due to the LASN vector used in TCGT (Table S2). The frequency of each integration was less biased in Pt2 than in Pt1.

### Biological influence of genes proximal to ISs on the engraftment of gene-marked cells

To assess the potential biological impact of vector integration on engraftment, we classified the genes proximal to the vector ISs into defined biological categories using the gene ontology database. Genes with read numbers of more than 0.1% of all integrations were analyzed to determine whether these were enriched in specific categories, but there were no statistically significant enriched categories in either patient (Figure S2).

We also analyzed the frequencies of the categories containing genes hit by the retroviral vector because the genetic/chromatin state of the cells at transduction could influence the insertion profiles.^14^ The genes hit in Pt1 and Pt2, which had multiple reads in NGS, were analyzed for categories relating to immune and hematopoietic systems (Table S3). Pt2 showed a relatively high frequency of genes involved in the immune system, including differentiation and response, compared to Pt1, which might reflect the transduction of peripheral T cells at TCGT. In contrast, Pt1 showed increased frequencies of genes with hematopoietic functions, which may be strongly associated with the engraftment of SCGT-derived cells in the BM (Figure S3).

Some genes with high read numbers were categorized as annotated cancer genes in both patients. In Pt1, almost one-half of the total reads was accounted for by integrations into *LOC100130950* (24.4%) and *TRAP1* (24.0%). *TRAP1* is involved in TNF receptor-mediated signal transduction, and overexpression of *TRAP1* decreases the production of reactive oxygen species, which accelerates the proliferation of tumor cells.^15^^,^^16^ Pt1 also showed two ISs near *GPX1* and *RAP1B* that have been reported to be oncogenes, with total read frequencies of 3.7% and 1.5%, respectively (Figure 3B). In Pt2, five integrations with total frequencies of more than 1% were observed near or in cancer-related genes (*DPP4*, *TNFAIP3/PERP*, *MLLT10*, *EPS8*, and *SPINT1*).

To determine the effect of these integrations on the expansion of the clones, we analyzed three of these genes with high frequencies for their expression levels (Figure 3C). The PB of Pt1 showed increased expression of these genes, and statistically significant enrichment was observed for *GPX1* and *RAP1B*. One integration located approximately 39 kb upstream of the *LMO2* gene was also analyzed; however, the expression of *LMO2* was not detected (data not shown). In Pt2, one integration located between two cancer-related genes, *TNFAIP3* and *PERP*, yielded increased expression of both genes with statistical significance; however, this integration was due to LASN, indicating integration only in peripheral T cells (Figure S4). Among the SCGT-derived clones with GCsapM-ADA integration, *SMARCC1* expression was also analyzed, although it has not been categorized as a cancer gene. Despite the high frequency of integration into *SMARCC1* in the IS analysis, we did not observe an increase in its expression level.

### Quantification of vector integrations in hematopoietic subpopulations

**Figure 4:**
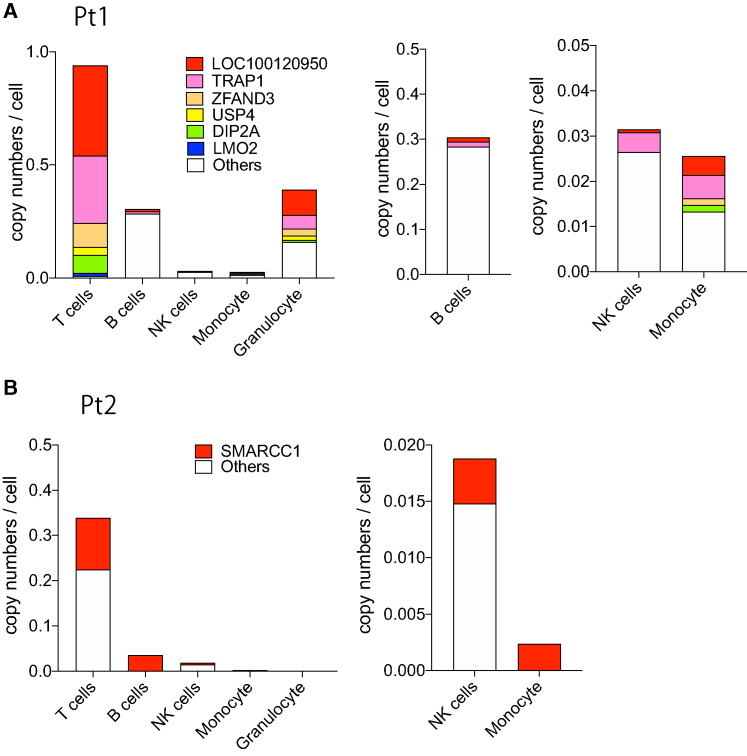
Engraftment of clones with vector integration into/near cancer-related genes (A) IS-specific ddPCR on integrations with high read numbers in Pt1. Five integrations, which had high read numbers in the LTR capture followed by high-throughput sequencing, were tracked in the hematopoietic subsets. The vector integration almost 39 kb upstream of the transcriptional start site of the *LMO2* gene was also analyzed. Enlarged figures on the integrations in B cells, NK cells, and monocytes are also shown. (B) The frequency of integration into *SMARCC1* in Pt2. Among the GCsapM-ADA integrations, clonal proliferation of the clone with integration into *SMARCC1* was observed. Integrations in NK cells and monocytes are also displayed on an enlarged scale.

We then investigated the presence of selected integrations that ranked high in read numbers in various hematopoietic lineages. Based on the sequences obtained via NGS, we designed primers/probes against the boundary between the vector and the genomic sequences and performed IS-specific ddPCR in T cells, B cells, NK cells, monocytes, and granulocytes. Pt1 showed integrations into *LOC100130950* and *TRAP1* in all lineages and three other integrations in T cell and myeloid-cell lineages (Figure 4A; Table 1). Integration upstream of LMO2 was detected only in T cells, with a copy number of 0.012 per cell. These results suggest the engraftment of a small population of vector-marked HSC/HPCs or less primitive progenitors with the potential for multi-lineage differentiation in Pt1. Quantification revealed that two integrations into *LOC100130950* and *TRAP1* comprised most of the vector integrations in the T cells. These two integrations also exhibited a slight dominance over other integrations in monocytes and granulocytes but not in B cells and NK cells. In Pt2, the integrations due to LASN were detected only in T cells, and the distribution of GCsapM-ADA integration into *SMARCC1* was explored. T cells showed a dominance of integration into *SMARCC1*, and this also comprised most of the integrations in B cells (Figure 4B).

**Table 1:** Integration site-specific droplet digital PCR for the integration of the GCsapM-ADA vector

		Copy number^a^				
	Gene	CD3^+^ T	CD19^+^ B	CD56^+^ NK	Mono	Gra
Pt1	*LOC100130950*	0.3989	0.0102	0.0007	0.0042	0.1124
	*TRAP1*	0.2978	0.0105	0.0043	0.0052	0.0610
	*ZFAND3*	0.1072	0	0	0.0014	0.0304
	*USP4*	0.0344	0	0	0	0.0193
	*DIP2A*	0.0806	0	0	0.0015	0.0097
	*LMO2*	0.0119	0	0	0	0
Pt2	*SMARCC1*	0.1137	0.0355	0.0040	0.0024	0

Pt1, patient 1; Pt2, patient 2; Mono, monocytes; Gra, granulocytes.

a Copy number was calculated as the number of signals per cell.

Whether these vector integrations facilitate the proliferation of clones remains unclear because these genes in the dominant clones (*LOC100130950* and *TRAP1* in Pt1 and *SMARCC1* in Pt2) were not categorized as cancer genes, or their expression levels did not increase. Each integration was analyzed for the distance from the active transcriptional start sites (TSS) of the nearest cellular gene. Unlike the typical pattern of retroviral vector,^14^ the integrations in both patients showed no tendency to accumulate at TSS of cellular genes (Figure S5). In contrast, three dominant integrations (*LOC100130950*, *TRAP1*, and *SMARCC1*) were in the active TSS of these genes, which could yield a higher level of vector transcription than other integrations (Figure S6). High ADA expression might facilitate the proliferation in the metabolically active subsets such as T cells, leading to the clonal dominance of these clones.

These results indicate that some factors other than insertional mutagenesis including the expression level of ADA in each clone may affect the clonal distributions of retrovirally transduced clones in ADA-SCID.

### Microenvironment characteristics of the BM

**Figure 5:**
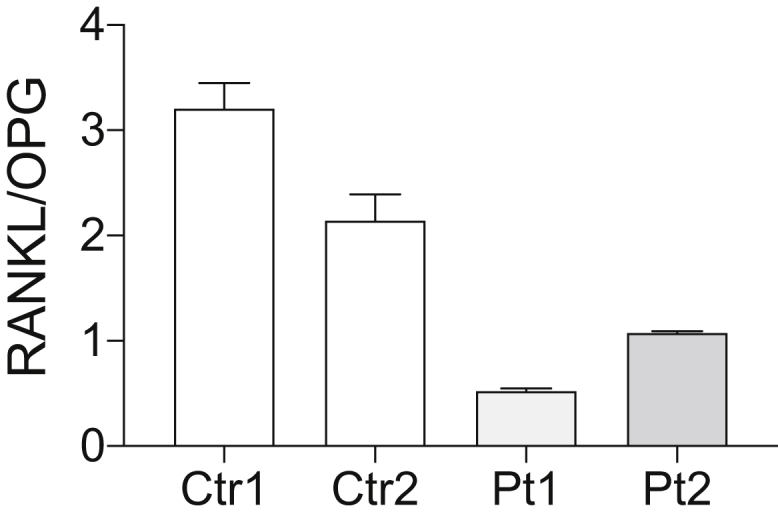
Reduced RANKL/OPG ratio in both patients Plasma samples from the patients were analyzed. Both patients showed a low RANKL/OPG ratio even after SCGT. A lower RANKL/OPG ratio observed in Pt1 than in Pt2 implied a severe defect in the BM microenvironment in Pt1.

Pt1 showed multi-lineage engraftment of vector-marked cells with a higher VCN in CD34-positive cells than that in Pt2. Pt1 displayed a severe clinical phenotype, and the accumulation of toxic metabolites might lead to impairment in the BM microenvironment, which may play a role as “auto-conditioning” and yield the engraftment of vector-marked progenitor cells without preconditioning therapy. Osteoblasts (OBs) and osteoclasts (OCs) are crucial components of the HSC niche and maintain stem cell properties, including self-renewal and multi-lineage hematopoiesis.^17^, ^18^, ^19^ Receptor activator of nuclear factor-κΒ ligand (RANKL) is produced by OBs and is required for crosstalk between OBs and OCs. The ratios of RANKL relative to its decoy receptor osteoprotegerin (OPG) were decreased in ADA-SCID patients,^20^ and SCGT recovers the microenvironment with an increase in RANKL level. We measured the ratios of RANKL to OPG in the plasma of both patients. Pt1 showed a lower RNAKL/OPG ratio than did Pt2, despite the engraftment of gene-corrected cells in the BM, indicating a severe defect in the BM microenvironment in Pt1 compared to Pt2 (Figure 5).

## Discussion

The patients in this study did not receive busulfan conditioning, and, therefore, full engraftment of gene-corrected cells was not successful. Several factors, including the disease background, influence the complex engraftment pattern of gene-corrected cells. A distribution of gene-marked cells revealed differences in the required levels of ADA in different cell types and at various differentiation stages. T cells are more sensitive to toxic metabolites than other immune cell subpopulations, so ADA activity is higher in T cells than in other cell types.^21^, ^22^, ^23^, ^24^, ^25^ Consistently, the patients in this study showed higher VCNs in T cells, indicating an increased need for ADA, than in other hematopoietic cells. Among T cells, homeostatic proliferation is higher in CD8^+^ T cells than in CD4^+^ T cells,^26^^,^^27^ and memory T cells appear to have higher metabolic activity than the naive subset.^28^^,^^29^ These features were reflected in the VCN pattern in Pt2, who had both transduced and non-transduced cells in the T cell subset. In Pt2, the VCN was higher in CD8^+^ T cells than in CD4^+^ T cells and increased along with differentiation from naive to memory subsets, which indicates that vector-derived ADA could metabolize the accumulated deoxyadenosine in memory subsets with frequent divisions.

The low VCNs in B cells, NK cells, and myeloid lineage cells suggest that vector-derived ADA did not confer a survival advantage in these cell types. Previous reports have shown that a selective advantage provided by ADA was observed in naive B cells but not in BM immature B cells.^30^ Although our patients showed a low VCN in naive B cells, the selective advantage of gene-corrected B cells could be observed during their maturation from naive to memory subsets, suggesting increasing levels of nucleic acid metabolism on B cell differentiation.

It has been reported that preferential targets for integration are closely related to the epigenetic state and expression profiles of the cell type at transduction.^14^ Therefore, the possible engraftment of vector-marked HPC/HSCs may reflect the integration profiles of Pt1 with a relatively high frequency of the ISs in or near genes involved in hematopoietic cell development, differentiation, and proliferation. In contrast, Pt2 possessed TCGT-derived cells in the periphery but showed few engraftments of the clones with multi-lineage differentiation potential. These engraftment patterns might correspond to the integration profiles of Pt2 with a relatively higher frequency of genes related to the immune system but a lower frequency related to the hematopoietic system than Pt1. However, the frequencies of genes with immune functions in Pt2 were lower than that in patients with PB lymphocyte-gene therapy in a previous report.^14^ More than 20 years passed since Pt2 received TCGT, so functional T cell clones with integration into immune system-related genes might have been exhausted by the time of our analysis.

Although neither patient developed leukemia in more than 10 years after SCGT, the impact of genetic alterations due to vector integrations on the long-term engraftment of vector-marked clones remains unclear. Both patients possessed clones with integrations near cancer-related genes. Pt1 showed the dominant proliferation of two clones, one of which had an integration into a cancer gene, *TRAP1*, but its expression level was not increased. In Pt2, LASN insertion between *TNFAIP3* and *PERP* caused the increased expression of both genes. Although transduction of peripheral T cells in TCGT did not cause the oncogenic transformation of repopulating cells, this integration might increase the long-term survival of this T cell clone. The integration into *SMARCC1* accounted for a large portion of GCsapM-ADA integrations, which indicates the clonal dominance of an SCGT-derived clone, also in Pt2. However, *SMARCC1* has not been reported as a cancer gene, and its expression level did not increase. These results suggest that mechanisms other than insertional mutagenesis induced the clonal proliferation of the dominant clones.

The dominant clones (*LOC100130950* and *TRAP1* in Pt1 and *SMARCC1* in Pt2) had GCsapM-ADA integrations in active TSS of cellular genes, which might result in effective transcription of vector-derived ADA. Most of other integrations in both patients, in contrast, were located away from the sites of H3K4me3 modification corresponding to TSS of the cellular genes and might yield a lower vector transcription. The hematopoietic subpopulations showed variable levels of ADA activity, and, therefore, a clone with a higher level of ADA activity could proliferate and dominate over other clones in metabolically active subsets including T cells. In B cells, Pt2 showed a clonal pattern of vector integrations, whereas Pt1 did not. Vector-derived ADA confers a growth advantage in memory B cells but not in naive B cells (Figure 2C), indicating that clonal dominance due to high ADA expression may be observed in memory B cells. Pt2 showed few integrations in naive B cells (VCN = 0.0025), and the possible clonal dominance in the memory subset may result in the clonal expansion of *SMARCC1* in the total B cell subset. In contrast, a relatively higher frequency of vector-marked cells in naive B cells (VCN = 0.049) might result in non-dominant proliferations of *LOC100130950* and *TRAP1* in total B cells of Pt1. In any case, these results suggest that the retroviral transduction of stem/progenitor cells yielded clonal hematopoiesis by clones with strong proliferating potential, which might be the result of various factors, in ADA-SCID.

Accumulation of toxic metabolites causes stem cell defects^31^ in ADA-SCID, which could play a role as auto-conditioning and create stem cell niches. Pt2 showed residual ADA activity in hematopoietic cell,s^11^ and there may be few niches in the BM, leading to lower engraftment of gene-corrected cells than that in Pt1. Toxic substrates may directly inhibit the survival of stem/progenitor cells, like other hematopoietic cells. Additionally, an abnormal environment in the BM may also reduce the number of stem cells.^19^ Sauer et al.^20^ reported that ADA-SCID patients showed a reduction in the RANKL/OPG ratio, indicating the OB insufficiency and impairment of the HSC niche, and SGCT rescued the microenvironment indicated by an increase in the RANKL/OPG ratio. Pt1 showed a lower RANKL/OPG ratio than did Pt2 despite a higher VCN in the BM, indicating that Pt1 had a severe defect in the BM microenvironment. It remains unclear whether the impaired BM microenvironment could provide a sufficient niche for the gene-corrected cells. However, the reduction in stem cells due to metabolic toxicity could have created space in the BM that facilitated engraftment without preconditioning.

Overall, SCGT without preconditioning led to a complex engraftment pattern of vector-marked cells, which may be affected by nucleotide metabolism in hematopoietic subpopulations and the BM microenvironment in ADA-SCID. The clonal proliferation of vector-integrated clones, although there was no increase in the transcription of cellular genes, implies that other mechanisms of clonal dominance are at play rather than insertional mutagenesis.

## Materials and methods

### Study approval

All study protocols involving the participation of the patients were approved by the Ethics Committee of the National Center for Child Health and Development. PB and BM samples were obtained from both patients. The patients and their parents provided written, informed consent to comply with standard ethical procedures.

### Flow cytometry and fluorescent-activated cell sorting

Mononuclear cells from PB and BM cells were stained with the following antibodies (BioLegend, San Diego, CA, USA): fluorescein isothiocyanate (FITC) anti-CD3, APC anti-CD19, PE anti-CD56, and PerCP-Cy5.5 anti-CD14 for the isolation of T cells (CD3^+^), B cells (CD19^+^), NK cells (CD56^+^), and monocytes (CD14^+^); APC anti-CD3, APC-Cy7 anti-CD4, Hoechst Blue anti-CD8, FITC anti-T cell receptor (TCR) γδ, FITC anti-CD45RA, and PE anti-CD31 for the isolation of T cell subpopulations; Hoechst Blue anti-CD19, PE anti-CD27, and APC-Cy7 anti-IgM for the isolation of B cell subpopulations; and PE anti-CD34 for BM CD34^+^ cells. Sorting of objective subsets was performed on a BD FACSAria II instrument (BD Biosciences, Franklin Lakes, NJ, USA).

### IS analysis

Genomic DNA was extracted from nucleated cells from the PB and BM samples. Genomic DNA (1,800 ng for Pt1 and 1,000 ng for Pt2) was fragmented using a focused ultrasonicator (Covaris M220), ligated with adaptors, and amplified using five to eight cycles of PCR using SureSelect XT Reagents (Agilent, Santa Clara, CA, USA) for Pt1 and the KAPA HyperPrep Kit (Kapa Biosystems, Wilmington, MA, USA) for Pt2, followed by purification using Agencourt AMPure XP beads (Beckman Coulter, Brea, CA, USA). The resultant pre-capture libraries (750 ng each) were hybridized with custom biotin-labeled capture RNA oligos designed against the vector LTR sequence for the target and the coding exons of the sonic hedgehog gene for hybridization controls (Data S1). Hybridized DNA was captured by streptavidin-coated beads and was then amplified using 15 cycles of PCR to add an index tag and adaptor sequences compatible with Illumina sequencing. Hybridization wash and post-capture amplification were conducted using SureSelect XT Reagents (Agilent) according to the manufacturer’s instructions. High-throughput sequencing was performed using the HiSeq 2500 system to generate paired-end reads (2 × 100 bp). Approximately 20 million and 40 million read pairs were obtained for the post-capture libraries for Pt1 and Pt2, respectively. The adaptor sequences of the sequencing reads were trimmed using cutadapt-2.1, and the low-quality bases at the read ends were removed using a custom script followed by mapping to the human reference genome (hs37d5) using BWA-0.7.13 with the entire vector sequence (GCsapM-ADA, 3,616 bp; LASN, 4,286 bp, containing the LTR sequences used as capture targets). PCR duplicates were removed using Picard-tools-2.1.1. Sequence reads with one end mapped to the vector and the other end mapped to the human genome were selected by a custom script, and a.bam file was created. The resultant.bam file was analyzed using the *FindCoveredIntervals* function of GenomeAnalysisTK-3.8 to make a list of ISs. The resultant list of ISs was annotated for neighboring genes using table_annovar.pl integrated into a custom script. Genes proximal to the IS were compared with a list of annotated cancer genes from the Atlas of Genetics and Cytogenetics in Oncology and Haematology database (http://www.atlasgeneticsoncology.org/).

### VCN analysis

Genomic DNA was extracted from sorted cell subsets, and the VCN was determined using the Bio-Rad QX200 ddPCR system (Bio-Rad, Hercules, CA, USA) with primers/probes directed against the vector packaging signal and the reference gene *RPP30*. The cell number was calculated as one-half of the *RPP30*-positive droplet counts, as each cell is diploid. The VCN was calculated as the number of vector copies per cell (see Data S2 for all primer/probe sequences). For Pt2, the copy number of the GCsapM-ADA vectors was also determined using primers and probe specific for the vector.

### IS-specific ddPCR

Primers and probes were designed to detect the boundaries between the LTR and the host genome in high-ranked integrations identified by high-throughput sequencing (Data S2). Genomic DNA from the target cell subsets was analyzed for the presence of each integration by ddPCR. The copy number of each integration was normalized by the cell number, which was calculated as one-half of the *RPP30*-positive droplets.

### Transcription levels of cancer-related genes proximal to ISs

RNA was extracted from the PB using the RNeasy Mini Kit (QIAGEN). Transcription levels of *TRAP1*, *GPX1*, and *RAP1B* in Pt1 and *DPP4*, *PERP*, *TNFAIP3*, *MLLT10*, and *SMARCC1* in Pt2 and the reference gene *TBP* were analyzed using the Prime Time Std qPCR Assay (Integrated DNA Technologies, Coralville, IA, USA) and One-Step RT-ddPCR Advanced Kit for Probes (Bio-Rad), followed by the calculation of signal-positive droplets using the Bio-Rad QX200 system. The expression level of each gene was normalized relative to the expression of *TBP*.

### ELISA assays of RANKL and OPG

An ELISA assay of RANKL and OPG was performed on plasma from patients and normal pediatric donors using Human RANKL ELISA kit and Human Osteoprotegerin ELISA kit (Abcam, Cambridge, UK) was performed on plasma from patients and normal pediatric donors according to the manufacturer’s instructions.

## Supplemental information

Document S1. Tables S1–S3, Figures S1–S6, and Data S1 and S2

Document S2. Article plus supplemental information
