# Supplementary material for: Nonconditioned ADA-SCID gene therapy reveals ADA requirement in the hematopoietic system and clonal dominance of vector-marked clones
Source: Mol Ther Methods Clin Dev. 2021 Oct 16;23:424–33. doi: 10.1016/j.omtm.2021.10.003 (PMC8566957; doi:10.1016/j.omtm.2021.10.003)
Supplement: Document S1. Tables S1–S3, Figures S1–S6, and Data S1 and S2 [file mmc1.pdf]

OMTM, Volume *23*

Supplemental information

Nonconditioned ADA-SCID gene therapy reveals ADA requirement in the hematopoietic system and clonal dominance of vector-marked clones

Toru Uchiyama, Sirirat Takahashi, Kazuhiko Nakabayashi, Kohji Okamura, Kaori Edasawa, Masafumi Yamada, Nobuyuki Watanabe, Emi Mochizuki, Toru Yasuda, Akane Miura, Motohiro Kato, Daisuke Tomizawa, Makoto Otsu, Tadashi Ariga, and Masafumi Onodera

Table S1

Vector integration sites in Pt1

Chr	Start	End	Name	Forward	Reverse	Total	Position	Gene	frequency	%
17	5095711	5096488	17_5096127	2592	1550	4142	ncRNA_intronic	LOC100130950	0.244077784	24.40777843
16	3767780	3768780	16_3768241	2725	1354	4079	upstream	TRAP1	0.240365351	24.03653506
6	37904786	37905347	6_37905100	501	517	1018	intronic	ZFAND3	0.059988214	5.99882145
21	47970900	47971979	21_47971480	579	376	955	intronic	DIP2A	0.056275781	5.627578079
3	49378913	49379740	3_49379404	353	280	633	intergenic	USP4(dist=1869),GPX1(dist=15185)	0.03730112	3.730111962
6	35015735	35016309	6_35016017	141	287	428	intronic	ANKS1A	0.025220978	2.52209782
19	18168215	18168632	19_18168419	201	129	330	intergenic	ARRDC2(dist=43509),IL12RB1(dist=1932)	0.019446081	1.944608132
3	194874136	194874470	3_194874309	145	157	302	intronic	XXYLT1	0.017796111	1.779611078
2	149807361	149807928	2_149807734	141	139	280	intronic	KIF5C	0.016499705	1.649970536
12	68773489	68773967	12_68773724	124	125	249	intergenic	MDM1(dist=47564),RAP1B(dist=230875)	0.014672952	1.467295227
12	26605144	26605894	12_26605513	81	105	186	intronic	ITPR2	0.010960519	1.096051856
20	47835222	47835722	20_47835486	38	109	147	upstream	DDX27	0.008662345	0.866234532
20	3994461	3994844	20_3994800	110	20	130	intronic	RNF24	0.007660577	0.766057749
16	74439800	74440130	16_74439975	34	91	125	intergenic	LOC283922(dist=37823),CLEC18B(dist=2534)	0.00736594	0.736593989
1	120929618	120930076	1_120929853	85	36	121	intronic	FCGR1B	0.00713023	0.713022982
8	29605007	29605457	8_29605250	11	98	109	ncRNA_intronic	LINC00589	0.0064231	0.642309959
16	70223212	70223542	16_70223369	71	31	102	intergenic	CLEC18C(dist=2572),LOC100506060(dist=30095)	0.006010607	0.601060695
11	33952676	33952835	11_33952722	17	84	101	intergenic	LMO2(dist=38887),CAPRIN1(dist=120488)	0.005951679	0.595167943
17	58469949	58470406	17_58470175	54	45	99	upstream	USP32	0.005833824	0.58338244
11	46141548	46141828	11_46141784	98	0	98	intronic	PHF21A	0.005774897	0.577489688
3	105080503	105080921	3_105080720	50	42	92	intergenic	MIR548A3(dist=1134616),ALCAM(dist=4817)	0.005421332	0.542133176
16	46390161	46390353	16_46390309	40	43	83	intergenic	NONE(dist=NONE),ANKRD26P1(dist=112920)	0.004890984	0.489098409
22	29785630	29785757	22_29785676	15	68	83	intergenic	AP1B1(dist=1105),RFPL1S(dist=47308)	0.004890984	0.489098409
5	40487066	40487489	5_40487287	39	41	80	intergenic	DAB2(dist=1061953),PTGER4(dist=192725)	0.004714202	0.471420153
3	128949356	128949857	3_128949402	10	68	78	intergenic	CNBP(dist=46593),COPG1(dist=19031)	0.004596346	0.459634649
16	46433364	46433556	16_46433410	35	36	71	intergenic	NONE(dist=NONE),ANKRD26P1(dist=69819)	0.004183854	0.418385386
17	19016484	19016831	17_19016671	28	43	71	intergenic	GRAP(dist=66336),GRAPL(dist=14091)	0.004183854	0.418385386
16	70000299	70000602	16_70000463	55	13	68	intergenic	CLEC18A(dist=2214),PDXDC2P(dist=9719)	0.004007071	0.40070713
20	43248885	43249028	20_43248931	18	46	64	exonic	ADA	0.003771361	0.377136123
17	18967998	18968345	17_18968177	26	25	51	intergenic	GRAP(dist=17842),GRAPL(dist=62585)	0.003005303	0.300530348
11	120424782	120424974	11_120424828	9	37	46	intergenic	ARHGEF12(dist=64184),GRIK4(dist=106180)	0.002710666	0.271066588
15	60269718	60269910	15_60269764	7	39	46	intergenic	BNIP2(dist=288123),FOXB1(dist=26637)	0.002710666	0.271066588
8	75623280	75623472	8_75623428	45	0	45	ncRNA_intronic	FLJ39080	0.002651738	0.265173836
17	18964343	18964690	17_18964513	35	7	42	intergenic	GRAP(dist=14178),GRAPL(dist=66249)	0.002474956	0.24749558
16	46396262	46396454	16_46396410	33	8	41	intergenic	NONE(dist=NONE),ANKRD26P1(dist=106819)	0.002416028	0.241602829
1	149760403	149760594	1_149760449	3	35	38	intronic	FCGR1A,HIST2H2BF	0.002239246	0.223924573
X	16973115	16973307	X_16973161	0	37	37	intronic	REPS2	0.002180318	0.218031821
1	149375481	149375673	1_149375527	1	35	36	ncRNA_intronic	FCGR1C	0.002121391	0.212139069
5	44438231	44438423	5_44438277	32	1	33	intergenic	FGF10(dist=49494),MRPS30(dist=370730)	0.001944608	0.194460813
9	140481099	140481291	9_140481145	27	4	31	intronic	ZMYND19	0.001826753	0.182675309
17	19090562	19090794	17_19090608	0	30	30	intergenic	GRAPL(dist=28461),EPN2(dist=50062)	0.001767826	0.176782557
5	61573109	61573331	5_61573287	25	0	25	intergenic	C5orf64(dist=570926),KIF2A(dist=28682)	0.001473188	0.147318798
5	170107817	170108009	5_170107863	8	17	25	intronic	KCNIP1	0.001473188	0.147318798
8	34892624	34892816	8_34892772	16	9	25	intergenic	DUSP26(dist=1435334),UNC5D(dist=200183)	0.001473188	0.147318798
20	43249613	43249833	20_43249659	0	25	25	exonic	ADA	0.001473188	0.147318798
21	19803579	19803771	21_19803727	16	8	24	intergenic	TMPRSS15(dist=27758),NONE(dist=NONE)	0.00141426	0.141426046
4	71511120	71511312	4_71511166	12	11	23	UTR3	ENAM	0.001355333	0.135533294
13	100123820	100124099	13_100124055	23	0	23	intergenic	MIR548AN(dist=65502),TM9SF2(dist=29553)	0.001355333	0.135533294
10	31020513	31020705	10_31020661	19	3	22	intergenic	LYZL2(dist=102015),ZNF438(dist=112884)	0.001296405	0.129640542
15	33206953	33207145	15_33206999	3	19	22	intronic	FMN1	0.001296405	0.129640542
7	78602613	78602805	7_78602761	21	0	21	intronic	MAGI2	0.001237478	0.12374779
7	122039169	122039361	7_122039317	11	10	21	intronic	CADPS2	0.001237478	0.12374779
1	246746379	246746571	1_246746425	11	7	18	intronic	CNST	0.001060695	0.106069534
2	222782484	222782676	2_222782632	6	12	18	intergenic	EPHA4(dist=345623),PAX3(dist=281954)	0.001060695	0.106069534
8	51983152	51983344	8_51983198	7	11	18	intergenic	SNTG1(dist=277772),PXDNL(dist=248919)	0.001060695	0.106069534
15	89831906	89832098	15_89831952	0	18	18	intronic	FANCI	0.001060695	0.106069534
16	70744461	70744653	16_70744609	18	0	18	intronic	VAC14	0.001060695	0.106069534
3	143478120	143478312	3_143478268	14	3	17	intronic	SLC9A9	0.001001768	0.100176783
3	145577166	145577358	3_145577314	10	7	17	intergenic	C3orf58(dist=1866105),PLOD2(dist=209894)	0.001001768	0.100176783
6	115531994	115532186	6_115532040	0	17	17	intergenic	HS3ST5(dist=1148000),FRK(dist=730633)	0.001001768	0.100176783
11	72769362	72769554	11_72769408	6	11	17	intronic	FCHSD2	0.001001768	0.100176783
14	66337094	66337286	14_66337242	17	0	17	intergenic	FUT8(dist=126404),LINC00238(dist=615847)	0.001001768	0.100176783
16	76802991	76803183	16_76803037	0	17	17	intergenic	CNTNAP4(dist=209903),MIR4719(dist=99776)	0.001001768	0.100176783
17	19094093	19094325	17_19094281	17	0	17	intergenic	GRAPL(dist=32134),EPN2(dist=46389)	0.001001768	0.100176783
2	55379913	55380105	2_55380061	2	14	16	intergenic	RTN4(dist=102328),CLHC1(dist=19606)	0.00094284	0.094284031
4	69733799	69733991	4_69733845	3	13	16	intergenic	UGT2B10(dist=36105),UGT2A3(dist=60312)	0.00094284	0.094284031
6	73220695	73220887	6_73220843	16	0	16	intergenic	RIMS1(dist=107999),KCNQ5(dist=110708)	0.00094284	0.094284031
16	7825688	7825880	16_7825836	16	0	16	intergenic	RBFOX1(dist=62497),TMEM114(dist=793646)	0.00094284	0.094284031
1	212477517	212477709	1_212477563	0	15	15	intronic	PPP2R5A	0.000883913	0.088391279
11	76793099	76793291	11_76793247	15	0	15	intronic	CAPN5	0.000883913	0.088391279
13	43122822	43123014	13_43122970	15	0	15	intergenic	AKAP11(dist=225568),TNFSF11(dist=13882)	0.000883913	0.088391279
15	35062371	35062563	15_35062417	0	15	15	intergenic	GJD2(dist=15636),ACTC1(dist=17860)	0.000883913	0.088391279
19	8466341	8466533	19_8466489	15	0	15	intronic	RAB11B	0.000883913	0.088391279
1	244905536	244905683	1_244905639	14	0	14	intergenic	DESI2(dist=33306),COX20(dist=92980)	0.000824985	0.082498527
6	170943748	170943940	6_170943896	14	0	14	intergenic	PDCD2(dist=50117),NONE(dist=NONE)	0.000824985	0.082498527
11	63312882	63313074	11_63313030	10	4	14	ncRNA_intronic	MIR3680-1,MIR3680-2	0.000824985	0.082498527
11	101949567	101949759	11_101949613	0	14	14	intronic	C11orf70	0.000824985	0.082498527
14	24912400	24912592	14_24912446	1	13	14	upstream	SDR39U1	0.000824985	0.082498527
15	42089187	42089379	15_42089233	0	14	14	intronic	MAPKBP1	0.000824985	0.082498527
4	56801000	56801192	4_56801046	0	13	13	intergenic	EXOC1(dist=29803),CEP135(dist=13908)	0.000766058	0.076605775
5	53559514	53559706	5_53559560	0	13	13	intronic	ARL15	0.000766058	0.076605775
5	144665206	144665398	5_144665252	0	13	13	intergenic	KCTD16(dist=808309),PRELID2(dist=473310)	0.000766058	0.076605775
6	107728174	107728366	6_107728220	0	13	13	intronic	PDSS2	0.000766058	0.076605775
9	6820085	6820277	9_6820131	0	13	13	intronic	KDM4C	0.000766058	0.076605775
12	17956438	17956630	12_17956484	0	13	13	intergenic	MIR3974(dist=130157),RERGL(dist=277299)	0.000766058	0.076605775
13	78271236	78271428	13_78271384	13	0	13	stream;downstrea	SLAIN1;MIR3665	0.000766058	0.076605775
15	40722633	40722825	15_40722781	13	0	13	intergenic	IVD(dist=9270),BAHD1(dist=10610)	0.000766058	0.076605775
16	84530648	84530840	16_84530796	8	5	13	intronic	TLDC1	0.000766058	0.076605775
3	4681289	4681481	3_4681437	12	0	12	intronic	ITPR1	0.00070713	0.070713023

3	87688937	87689129	3_87688983	0	12	12	intergenic	POU1F1(dist=363247),HTR1F(dist=342723)	0.00070713	0.070713023
5	32841705	32841897	5_32841853	6	6	12	intergenic	NPR3(dist=50024),LOC340113(dist=105676)	0.00070713	0.070713023
6	13620478	13620670	6_13620524	0	12	12	exonic	NOL7	0.00070713	0.070713023
2	30766812	30767004	2_30766960	11	0	11	intronic	LCLAT1	0.000648203	0.064820271
2	109937967	109938156	2_109938112	11	0	11	intronic	SH3RF3	0.000648203	0.064820271
7	93406849	93407041	7_93406997	1	10	11	intergenic	MIR4652(dist=60681),TFPI2(dist=107692)	0.000648203	0.064820271
9	79813944	79814136	9_79814092	11	0	11	intronic	VPS13A	0.000648203	0.064820271
10	58422463	58422655	10_58422509	0	11	11	intergenic	ZWINT(dist=301476),MIR3924(dist=641710)	0.000648203	0.064820271
14	50057673	50057865	14_50057821	11	0	11	intergenic	RPS29(dist=4688),LRR1(dist=7574)	0.000648203	0.064820271
1	143289385	143289577	1_143289431	0	10	10	intergenic	RD20A12P(dist=575827),LOC100130000(dist=397	0.000589275	0.058927519
2	8008952	8009143	2_8008998	0	10	10	intergenic	OC100506274(dist=418618),LOC339788(dist=5353	0.000589275	0.058927519
3	2727201	2727393	3_2727247	0	10	10	intronic	CNTN4	0.000589275	0.058927519
4	184003734	184003926	4_184003882	10	0	10	intergenic	FAM92A1P2(dist=42611),WWC2-AS2(dist=14272)	0.000589275	0.058927519
5	9785443	9785635	5_9785591	10	0	10	ncRNA_intronic	LOC285692	0.000589275	0.058927519
5	156376265	156376457	5_156376311	1	9	10	intronic	TIMD4	0.000589275	0.058927519
6	31509379	31509571	6_31509425	0	10	10	ncRNA_intronic	ATP6V1G2-DDX39B	0.000589275	0.058927519
6	106448487	106448679	6_106448533	0	10	10	intergenic	PREP(dist=597535),PRDM1(dist=85642)	0.000589275	0.058927519
9	17939245	17939437	9_17939291	0	10	10	intergenic	SH3GL2(dist=142170),ADAMTSL1(dist=534768)	0.000589275	0.058927519
13	79551510	79551702	13_79551658	10	0	10	intergenic	LINC00331(dist=137474),RBM26(dist=342422)	0.000589275	0.058927519
17	57914420	57914612	17_57914568	10	0	10	intronic	VMP1	0.000589275	0.058927519
18	43568391	43568583	18_43568437	0	10	10	intronic	PSTPIP2	0.000589275	0.058927519
21	17082868	17083060	21_17082914	0	10	10	intergenic	NRIP1(dist=645789),USP25(dist=19562)	0.000589275	0.058927519
21	46703416	46703608	21_46703462	0	10	10	intronic	POFUT2	0.000589275	0.058927519
1	218176686	218176878	1_218176732	0	9	9	intergenic	LINC00210(dist=82587),RRP15(dist=281877)	0.000530348	0.053034767
4	37358618	37358810	4_37358664	0	9	9	intronic	KIAA1239	0.000530348	0.053034767
5	127926877	127927069	5_127926923	0	9	9	intergenic	FBN2(dist=53189),SLC27A6(dist=374267)	0.000530348	0.053034767
6	96280620	96280812	6_96280666	0	9	9	intergenic	MANEA(dist=223339),FUT9(dist=183159)	0.000530348	0.053034767
6	105583167	105583359	6_105583315	9	0	9	intronic	BVES	0.000530348	0.053034767
11	21940617	21940809	11_21940663	0	9	9	intergenic	NELL1(dist=343435),ANO5(dist=274039)	0.000530348	0.053034767
11	64314135	64314327	11_64314283	9	0	9	intergenic	LOC100996455(dist=95156),SLC22A11(dist=8795	0.000530348	0.053034767
12	101207386	101207578	12_101207534	9	0	9	intronic	ANO4	0.000530348	0.053034767
14	98167889	98168081	14_98168037	9	0	9	intergenic	.OC100129345(dist=15043),C14orf64(dist=22389	0.000530348	0.053034767
1	16248218	16248410	1_16248366	8	0	8	intronic	SPEN	0.00047142	0.047142015
2	229672371	229672563	2_229672417	0	8	8	intergenic	SPHKAP(dist=626057),PID1(dist=216252)	0.00047142	0.047142015
3	161224757	161224949	3_161224803	0	8	8	intergenic	OTOL1(dist=3074),CT64(dist=1670208)	0.00047142	0.047142015
4	45134244	45134436	4_45134290	0	8	8	intergenic	GNPDA2(dist=405640),GABRG1(dist=903477)	0.00047142	0.047142015
5	90564964	90565156	5_90565010	0	8	8	intergenic	GPR98(dist=104978),ARRDC3(dist=99511)	0.00047142	0.047142015
5	169810558	169810750	5_169810706	5	3	8	exonic	KCNMB1	0.00047142	0.047142015
7	45066294	45066486	7_45066442	8	0	8	intronic	CCM2	0.00047142	0.047142015
7	63849337	63849529	7_63849485	8	0	8	intergenic	ZNF736(dist=39469),LOC649395(dist=44566)	0.00047142	0.047142015
8	24547411	24547603	8_24547457	0	8	8	intergenic	ADAM7(dist=180381),NEFM(dist=223797)	0.00047142	0.047142015
9	71197855	71198047	9_71197901	0	8	8	intergenic	TMEM252(dist=42119),PIP5K1B(dist=122695)	0.00047142	0.047142015
13	42249618	42249807	13_42249664	0	8	8	intronic	VWA8	0.00047142	0.047142015
13	102615622	102615814	13_102615668	0	8	8	intronic	FGF14	0.00047142	0.047142015
15	87326184	87326376	15_87326230	0	8	8	intronic	AGBL1	0.00047142	0.047142015
17	56744877	56745069	17_56745025	6	2	8	intronic	TEX14	0.00047142	0.047142015
18	63292196	63292388	18_63292242	0	8	8	intergenic	LOC284294(dist=1201416),CDH7(dist=125226)	0.00047142	0.047142015
X	62434371	62434563	X_62434417	3	5	8	intergenic	NONE(dist=NONE),SPIN4(dist=132670)	0.00047142	0.047142015
X	92782156	92782348	X_92782202	0	8	8	intergenic	PCDH11X(dist=903975),NAP1L3(dist=143703)	0.00047142	0.047142015
1	55328081	55328273	1_55328229	7	0	7	intronic	DHCR24	0.000412493	0.041249263
1	86994872	86995064	1_86994918	0	7	7	intergenic	CLCA1(dist=28945),CLCA4(dist=17821)	0.000412493	0.041249263
1	189680129	189680321	1_189680175	0	7	7	intergenic	NONE(dist=NONE),FAM5C(dist=386602)	0.000412493	0.041249263
2	133306742	133306934	2_133306788	0	7	7	intronic	GPR39	0.000412493	0.041249263
2	171880918	171881110	2_171880964	0	7	7	intronic	TLK1	0.000412493	0.041249263
2	195913037	195913229	2_195913185	7	0	7	intergenic	NONE(dist=NONE),SLC39A10(dist=608327)	0.000412493	0.041249263
3	106002199	106002391	3_106002347	7	0	7	intergenic	CBLB(dist=414461),LOC100302640(dist=826270)	0.000412493	0.041249263
3	138913754	138913946	3_138913800	0	7	7	intergenic	BPESC1(dist=69796),PISRT1(dist=38014)	0.000412493	0.041249263
3	188893040	188893232	3_188893188	7	0	7	intronic	TPRG1	0.000412493	0.041249263
4	146655061	146655253	4_146655209	7	0	7	intergenic	C4orf51(dist=1262),ZNF827(dist=26659)	0.000412493	0.041249263
6	93977701	93977893	6_93977849	7	0	7	intronic	EPHA7	0.000412493	0.041249263
6	167248834	167249026	6_167248982	7	0	7	intronic	RPS6KA2	0.000412493	0.041249263
7	22595320	22595512	7_22595468	7	0	7	intergenic	STEAP1B(dist=55568),LOC100506178(dist=7468)	0.000412493	0.041249263
8	3598731	3598923	8_3598777	0	7	7	intronic	CSMD1	0.000412493	0.041249263
10	107858369	107858561	10_107858517	7	0	7	intergenic	SORCS3(dist=833525),SORCS1(dist=474884)	0.000412493	0.041249263
11	35525950	35526142	11_35525996	0	7	7	intronic	PAMR1	0.000412493	0.041249263
11	88185447	88185639	11_88185493	0	7	7	intergenic	CTSC(dist=114553),GRM5(dist=52231)	0.000412493	0.041249263
11	111711860	111712052	11_111712008	7	0	7	intronic	ALG9	0.000412493	0.041249263
12	24036204	24036396	12_24036352	7	0	7	intronic	SOX5	0.000412493	0.041249263
12	100550406	100550598	12_100550452	0	7	7	ncRNA_exonic	GOLGA2P5	0.000412493	0.041249263
13	27143432	27143624	13_27143478	0	7	7	intronic	WASF3	0.000412493	0.041249263
13	69719214	69719406	13_69719362	7	0	7	intergenic	LINC00550(dist=259906),KLHL1(dist=555343)	0.000412493	0.041249263
13	81899969	81900158	13_81900114	7	0	7	intergenic	SPRY2(dist=985029),NONE(dist=NONE)	0.000412493	0.041249263
14	34178408	34178600	14_34178454	2	5	7	intronic	NPAS3	0.000412493	0.041249263
17	13714142	13714334	17_13714188	0	7	7	intergenic	HS3ST3A1(dist=208945),CDRT15P1(dist=213607)	0.000412493	0.041249263
20	60584776	60584968	20_60584822	3	4	7	intronic	TAF4	0.000412493	0.041249263
2	175709084	175709276	2_175709232	6	0	6	intronic	CHN1	0.000353565	0.035356511
2	211181639	211181831	2_211181685	0	6	6	intergenic	MYL1(dist=1791),LANCL1(dist=114268)	0.000353565	0.035356511
3	53583272	53583464	3_53583420	6	0	6	intronic	CACNA1D	0.000353565	0.035356511
3	64899623	64899815	3_64899669	0	6	6	ncRNA_intronic	ADAMTS9-AS2,MIR548A2	0.000353565	0.035356511
3	144602171	144602363	3_144602319	6	0	6	intergenic	C3orf58(dist=891110),PLOD2(dist=1184889)	0.000353565	0.035356511
7	136205921	136206113	7_136205967	0	6	6	intergenic	MTPN(dist=543764),CHRM2(dist=347412)	0.000353565	0.035356511
8	32378389	32378581	8_32378537	6	0	6	intronic	NRG1	0.000353565	0.035356511
8	36160636	36160828	8_36160784	6	0	6	intergenic	UNC5D(dist=508604),KCNU1(dist=481038)	0.000353565	0.035356511
9	28744910	28745102	9_28745058	6	0	6	intronic	LINGO2	0.000353565	0.035356511
9	115314484	115314676	9_115314632	6	0	6	intronic	KIAA1958	0.000353565	0.035356511
9	129098784	129098976	9_129098932	6	0	6	intronic	MVB12B	0.000353565	0.035356511
11	19579187	19579379	11_19579233	0	6	6	intronic	NAV2	0.000353565	0.035356511
11	49555465	49555652	11_49555511	0	6	6	intergenic	FOLH1(dist=325290),LOC440040(dist=24549)	0.000353565	0.035356511
11	74507281	74507473	11_74507429	4	2	6	intronic	RNF169	0.000353565	0.035356511
12	67541148	67541340	12_67541194	0	6	6	intergenic	GRIP1(dist=468270),CAND1(dist=121847)	0.000353565	0.035356511
14	84830643	84830835	14_84830791	6	0	6	intergenic	NONE(dist=NONE),FLRT2(dist=1165677)	0.000353565	0.035356511
15	50916023	50916215	15_50916069	0	6	6	intronic	TRPM7	0.000353565	0.035356511
16	16967306	16967498	16_16967352	0	6	6	intergenic	PKD1P1(dist=522888),XYLT1(dist=228809)	0.000353565	0.035356511
17	3733360	3733552	17_3733508	6	0	6	intronic	C17orf85	0.000353565	0.035356511
17	32353342	32353534	17_32353490	6	0	6	intronic	ASIC2	0.000353565	0.035356511

18	73144576	73144768	18_73144622	0	6	6	intergenic	SMIM21(dist=5034),LOC339298(dist=690311)	0.000353565	0.035356511
X	8735797	8735989	X_8735843	0	6	6	intergenic	KAL1(dist=35617),FAM9A(dist=22974)	0.000353565	0.035356511
X	83913326	83913518	X_83913372	0	6	6	intergenic	HDX(dist=155886),UBE2DNL(dist=275765)	0.000353565	0.035356511
X	87676720	87676912	X_87676766	0	6	6	intergenic	KLHL4(dist=751717),CPXCR1(dist=325440)	0.000353565	0.035356511
1	22870098	22870290	1_22870246	5	0	5	intergenic	ZBTB40(dist=12597),EPHA8(dist=19738)	0.000294638	0.02946376
1	180391212	180391404	1_180391258	0	5	5	intronic	ACBD6	0.000294638	0.02946376
2	61767796	61767988	2_61767842	0	5	5	intergenic	XPO1(dist=2425),FAM161A(dist=284121)	0.000294638	0.02946376
2	99545089	99545281	2_99545135	3	2	5	intronic	KIAA1211L	0.000294638	0.02946376
3	83960564	83960753	3_83960610	0	5	5	intergenic	NONE(dist=NONE),LOC440970(dist=726926)	0.000294638	0.02946376
3	84340649	84340841	3_84340797	5	0	5	intergenic	NONE(dist=NONE),LOC440970(dist=346739)	0.000294638	0.02946376
3	135395898	135396090	3_135395944	0	5	5	intergenic	EPHB1(dist=416638),PPP2R3A(dist=288551)	0.000294638	0.02946376
4	20510731	20510923	4_20510879	5	0	5	intronic	SLIT2	0.000294638	0.02946376
4	141320454	141320646	4_141320602	5	0	5	intronic	CLGN	0.000294638	0.02946376
5	27745817	27746009	5_27745965	5	0	5	intergenic	LOC643401(dist=249458),LSP1P3(dist=1180992)	0.000294638	0.02946376
5	60879923	60880115	5_60879969	0	5	5	intergenic	ZSWIM6(dist=37971),C5orf64(dist=53647)	0.000294638	0.02946376
5	143399301	143399493	5_143399347	0	5	5	intergenic	HMHB1(dist=199064),YIPF5(dist=138356)	0.000294638	0.02946376
6	69086619	69086811	6_69086665	0	5	5	intergenic	NONE(dist=NONE),BAI3(dist=258947)	0.000294638	0.02946376
6	111008623	111008815	6_111008669	0	5	5	intronic	CDK19	0.000294638	0.02946376
10	23624816	23625008	10_23624964	4	1	5	intronic	C10orf67	0.000294638	0.02946376
11	100115411	100115603	11_100115559	5	0	5	intronic	CNTN5	0.000294638	0.02946376
12	65730913	65731105	12_65730959	0	5	5	intronic	MSRB3	0.000294638	0.02946376
13	85040395	85040587	13_85040441	0	5	5	ncRNA_intronic	LINC00333	0.000294638	0.02946376
16	4574670	4574862	16_4574716	0	5	5	intronic	CDIP1	0.000294638	0.02946376
16	26105616	26105808	16_26105662	0	5	5	intronic	HS3ST4	0.000294638	0.02946376
16	80164333	80164525	16_80164481	5	0	5	intergenic	MAF(dist=529860),DYNLRB2(dist=410353)	0.000294638	0.02946376
18	62496563	62496755	18_62496609	0	5	5	intergenic	LOC284294(dist=405783),CDH7(dist=920859)	0.000294638	0.02946376
21	22569847	22570039	21_22569893	0	5	5	intronic	NCAM2	0.000294638	0.02946376
1	51832180	51832372	1_51832328	4	0	4	intronic	EPS15	0.00023571	0.023571008
1	182252726	182252918	1_182252874	2	2	4	intergenic	ZNF648(dist=222028),GLUL(dist=97945)	0.00023571	0.023571008
1	213699493	213699685	1_213699641	4	0	4	intergenic	RPS6KC1(dist=252834),LINC00538(dist=398431)	0.00023571	0.023571008
2	52186636	52186828	2_52186682	0	4	4	intergenic	NRXN1(dist=927009),ASB3(dist=1710415)	0.00023571	0.023571008
2	75528913	75529105	2_75529061	4	0	4	intergenic	TACR1(dist=102417),EVA1A(dist=190363)	0.00023571	0.023571008
2	151762362	151762554	2_151762510	4	0	4	intergenic	RND3(dist=418302),RBM43(dist=342198)	0.00023571	0.023571008
2	176438676	176438868	2_176438824	4	0	4	intergenic	ATP5G3(dist=392335),KIAA1715(dist=351566)	0.00023571	0.023571008
2	176649435	176649627	2_176649583	4	0	4	intergenic	ATP5G3(dist=603094),KIAA1715(dist=140807)	0.00023571	0.023571008
2	240884665	240884857	2_240884813	4	0	4	intergenic	MIR4786(dist=2303),NDUFA10(dist=11956)	0.00023571	0.023571008
3	36280555	36280747	3_36280601	0	4	4	intergenic	ARPP21(dist=444614),STAC(dist=141476)	0.00023571	0.023571008
3	66308141	66308333	3_66308187	0	4	4	intronic	SLC25A26	0.00023571	0.023571008
3	76128838	76129030	3_76128884	2	2	4	intergenic	ZNF717(dist=294630),ROBO2(dist=960390)	0.00023571	0.023571008
4	64843	65035	4_64991	1	3	4	intronic	ZNF595,ZNF718	0.00023571	0.023571008
4	65439308	65439500	4_65439456	4	0	4	intergenic	TECRL(dist=164279),LOC401134(dist=340523)	0.00023571	0.023571008
4	75188710	75188902	4_75188756	0	4	4	intergenic	EPGN(dist=6237),EREG(dist=42084)	0.00023571	0.023571008
5	139280865	139281057	5_139281013	4	0	4	intronic	NRG2	0.00023571	0.023571008
5	165097728	165097920	5_165097876	4	0	4	intergenic	NONE(dist=NONE),TENM2(dist=1613947)	0.00023571	0.023571008
5	166737936	166738128	5_166737982	0	4	4	intronic	TENM2	0.00023571	0.023571008
6	70677966	70678158	6_70678012	0	4	4	intronic	COL19A1	0.00023571	0.023571008
6	82928997	82929189	6_82929145	4	0	4	intronic	IBTK	0.00023571	0.023571008
7	28294280	28294472	7_28294428	4	0	4	intergenic	JAZF1-AS1(dist=13433),CREB5(dist=44492)	0.00023571	0.023571008
7	29071432	29071624	7_29071478	0	4	4	intronic	CPVL	0.00023571	0.023571008
7	39274461	39274653	7_39274507	0	4	4	intronic	POU6F2	0.00023571	0.023571008
7	40509538	40509730	7_40509584	0	4	4	intronic	C7orf10	0.00023571	0.023571008
7	45130987	45131179	7_45131135	4	0	4	intergenic	NACAD(dist=2643),TBRG4(dist=8544)	0.00023571	0.023571008
7	51801243	51801435	7_51801289	0	4	4	intergenic	COBL(dist=416775),POM121L12(dist=1302040)	0.00023571	0.023571008
8	122704422	122704614	8_122704468	0	4	4	intergenic	HAS2-AS1(dist=46905),ZHX2(dist=1089413)	0.00023571	0.023571008
9	5548926	5549118	9_5548972	0	4	4	intronic	PDCD1LG2	0.00023571	0.023571008
12	63659838	63660030	12_63659884	0	4	4	intergenic	AVPR1A(dist=113295),DPY19L2(dist=292789)	0.00023571	0.023571008
15	102411217	102411409	15_102411365	4	0	4	intergenic	OR4F13P(dist=20839),OR4F4(dist=50960)	0.00023571	0.023571008
16	63570846	63571038	16_63570892	0	4	4	intergenic	CDH8(dist=1500154),CDH11(dist=1409771)	0.00023571	0.023571008
18	69991031	69991223	18_69991077	0	4	4	intergenic	OC100505776(dist=744886),CBLN2(dist=212818	0.00023571	0.023571008
19	27968150	27968343	19_27968196	0	4	4	intergenic	NONE(dist=NONE),LINC00662(dist=313185)	0.00023571	0.023571008
X	62383732	62383924	X_62383880	2	2	4	intergenic	NONE(dist=NONE),SPIN4(dist=183207)	0.00023571	0.023571008
X	109883266	109883458	X_109883312	0	4	4	intergenic	TDGF1P3(dist=117064),CHRDL1(dist=33752)	0.00023571	0.023571008
X	123855575	123855767	X_123855621	0	4	4	intronic	TENM1	0.00023571	0.023571008
1	117005966	117006158	1_117006114	3	0	3	intergenic	ATP1A1OS(dist=44871),CD58(dist=51022)	0.000176783	0.017678256
1	169742061	169742253	1_169742107	0	3	3	intergenic	SELE(dist=38888),METTL18(dist=19543)	0.000176783	0.017678256
1	207662124	207662316	1_207662170	0	3	3	intronic	CR2	0.000176783	0.017678256
2	61018852	61019044	2_61019000	3	0	3	exonic	PAPOLG	0.000176783	0.017678256
2	117543928	117544120	2_117543974	0	3	3	intergenic	DPP10(dist=941649),DDX18(dist=1028261)	0.000176783	0.017678256
2	123917324	123917516	2_123917472	3	0	3	intergenic	TSN(dist=1392045),CNTNAP5(dist=865372)	0.000176783	0.017678256
2	174023164	174023352	2_174023210	0	3	3	intronic	ZAK	0.000176783	0.017678256
3	61667662	61667854	3_61667810	3	0	3	intronic	PTPRG	0.000176783	0.017678256
3	132800963	132801155	3_132801009	0	3	3	intronic	TMEM108	0.000176783	0.017678256
5	13367510	13367702	5_13367658	3	0	3	intergenic	CT49(dist=562364),DNAH5(dist=322759)	0.000176783	0.017678256
5	68931581	68931773	5_68931729	2	1	3	intergenic	LOC100272216(dist=2702),GUSBP3(dist=3541)	0.000176783	0.017678256
5	165133169	165133361	5_165133317	3	0	3	intergenic	NONE(dist=NONE),TENM2(dist=1578506)	0.000176783	0.017678256
6	36374815	36375007	6_36374861	0	3	3	intronic	PXT1	0.000176783	0.017678256
6	130701686	130701878	6_130701834	3	0	3	intronic	TMEM200A	0.000176783	0.017678256
7	2750055	2750247	7_2750101	0	3	3	intronic	AMZ1	0.000176783	0.017678256
7	57817400	57817592	7_57817548	3	0	3	intergenic	ZNF716(dist=284284),NONE(dist=NONE)	0.000176783	0.017678256
8	8038458	8038650	8_8038504	0	3	3	intergenic	MIR548I3(dist=91894),FAM86B3P(dist=47568)	0.000176783	0.017678256
8	36169405	36169597	8_36169451	0	3	3	intergenic	UNC5D(dist=517271),KCNU1(dist=472371)	0.000176783	0.017678256
8	36832565	36832757	8_36832713	3	0	3	intergenic	KCNU1(dist=39071),ZNF703(dist=720568)	0.000176783	0.017678256
8	53379287	53379479	8_53379333	0	3	3	intergenic	ST18(dist=56895),FAM150A(dist=67244)	0.000176783	0.017678256
8	72738491	72738683	8_72738537	2	1	3	intergenic	EYA1(dist=464071),MSC(dist=15220)	0.000176783	0.017678256
9	15618834	15619026	9_15618982	3	0	3	intronic	CCDC171	0.000176783	0.017678256
9	20630622	20630814	9_20630668	0	3	3	intergenic	MLLT3(dist=8155),FOCAD(dist=27621)	0.000176783	0.017678256
9	43087667	43087859	9_43087713	0	3	3	intergenic	FAM95B1(dist=54402),ANKRD20A3(dist=2239)	0.000176783	0.017678256
9	76795803	76795995	9_76795849	0	3	3	intergenic	ANXA1(dist=1010543),RORB(dist=316383)	0.000176783	0.017678256
9	110337114	110337306	9_110337262	3	0	3	intergenic	KLF4(dist=85216),ACTL7B(dist=1279587)	0.000176783	0.017678256
9	117337057	117337249	9_117337205	3	0	3	intergenic	DFNB31(dist=69470),ATP6V1G1(dist=12769)	0.000176783	0.017678256
10	121833801	121833993	10_121833847	0	3	3	intergenic	MIR4682(dist=115744),PPAPDC1A(dist=382599)	0.000176783	0.017678256
11	76352096	76352288	11_76352244	3	0	3	intergenic	C11orf30(dist=89656),LRRC32(dist=16304)	0.000176783	0.017678256
11	80855630	80855822	11_80855778	3	0	3	intergenic	TENM4(dist=1704084),MIR4300(dist=745985)	0.000176783	0.017678256
11	112333982	112334174	11_112334028	0	3	3	intergenic	C11orf34(dist=202446),NCAM1(dist=497921)	0.000176783	0.017678256
11	112834000	112834192	11_112834046	2	1	3	intronic	NCAM1	0.000176783	0.017678256

12	3666580	3666772	12_3666626	0	3	3	intronic	PRMT8	0.000176783	0.017678256
12	81256145	81256337	12_81256191	0	3	3	intronic	LIN7A	0.000176783	0.017678256
13	73159582	73159774	13_73159628	1	2	3	intergenic	DACH1(dist=718299),MZT1(dist=122847)	0.000176783	0.017678256
14	37789689	37789881	14_37789735	0	3	3	intronic	MIPOL1	0.000176783	0.017678256
15	73322735	73322927	15_73322781	0	3	3	intergenic	ADPGK-AS1(dist=232242),NEO1(dist=22024)	0.000176783	0.017678256
15	88591908	88592100	15_88591954	0	3	3	intronic	NTRK3	0.000176783	0.017678256
17	34575340	34575532	17_34575386	0	3	3	intergenic	CCL4L2(dist=35113),TBC1D3C(dist=5679)	0.000176783	0.017678256
17	34740374	34740566	17_34740420	0	3	3	intergenic	CCL4L2(dist=98581),TBC1D3H(dist=5496)	0.000176783	0.017678256
17	36331966	36332158	17_36332012	0	3	3	intergenic	TBC1D3(dist=36915),TBC1D3(dist=5496)	0.000176783	0.017678256
17	37936383	37936567	17_37936429	0	3	3	intronic	IKZF3	0.000176783	0.017678256
19	33088321	33088513	19_33088469	3	0	3	UTR3	ANKRD27	0.000176783	0.017678256
19	57453208	57453400	19_57453254	0	3	3	intergenic	MIMT1(dist=93333),USP29(dist=178235)	0.000176783	0.017678256
20	30042965	30043157	20_30043113	3	0	3	intergenic	DEFB123(dist=5054),DEFB124(dist=10176)	0.000176783	0.017678256
22	29730559	29730751	22_29730605	0	3	3	intronic	AP1B1	0.000176783	0.017678256
X	56593404	56593596	X_56593450	0	3	3	downstream	UBQLN2	0.000176783	0.017678256
X	63277145	63277337	X_63277293	3	0	3	intergenic	MIR1468(dist=271327),AMER1(dist=127684)	0.000176783	0.017678256
X	141271340	141271532	X_141271488	3	0	3	intergenic	MAGEC1(dist=274302),MAGEC2(dist=18620)	0.000176783	0.017678256
1	45344833	45345022	1_45344879	0	2	2	intronic	EIF2B3	0.000117855	0.011785504
1	53759264	53759456	1_53759412	2	0	2	intronic	LRP8	0.000117855	0.011785504
1	103185672	103185864	1_103185820	2	0	2	intergenic	OLFM3(dist=723031),COL11A1(dist=156183)	0.000117855	0.011785504
2	108323494	108323686	2_108323540	0	2	2	intergenic	ST6GAL2(dist=819978),LOC729121(dist=115960)	0.000117855	0.011785504
2	157009441	157009633	2_157009589	2	0	2	intergenic	KCNJ3(dist=1294726),NR4A2(dist=171335)	0.000117855	0.011785504
2	164923762	164923954	2_164923910	2	0	2	intergenic	FIGN(dist=331398),GRB14(dist=425393)	0.000117855	0.011785504
3	118890629	118890821	3_118890675	0	2	2	intergenic	C3orf30(dist=20374),UPK1B(dist=1730)	0.000117855	0.011785504
3	170571569	170571757	3_170571713	2	0	2	intergenic	SLC7A14(dist=267851),RPL22L1(dist=10932)	0.000117855	0.011785504
4	90636357	90636549	4_90636505	2	0	2	intergenic	GPRIN3(dist=407345),SNCA(dist=8725)	0.000117855	0.011785504
5	70467484	70467676	5_70467632	2	0	2	intergenic	LOC647859(dist=78736),GUSBP9(dist=48401)	0.000117855	0.011785504
5	139711417	139711609	5_139711565	2	0	2	downstream	HBEGF	0.000117855	0.011785504
6	64036526	64036718	6_64036674	2	0	2	intergenic	LGSN(dist=6793),PTP4A1(dist=245226)	0.000117855	0.011785504
7	69738523	69738715	7_69738671	2	0	2	intronic	AUTS2	0.000117855	0.011785504
7	83941191	83941380	7_83941336	2	0	2	intergenic	SEMA3A(dist=117120),SEMA3D(dist=683516)	0.000117855	0.011785504
8	48992908	48993100	8_48993056	2	0	2	intergenic	UBE2V2(dist=18603),EFCAB1(dist=634398)	0.000117855	0.011785504
8	144055898	144056090	8_144055944	0	2	2	intergenic	CYP11B2(dist=56686),LOC100133669(dist=7484)	0.000117855	0.011785504
9	77166094	77166286	9_77166140	0	2	2	intronic	RORB	0.000117855	0.011785504
9	80560666	80560858	9_80560712	0	2	2	intronic	GNAQ	0.000117855	0.011785504
9	88491327	88491519	9_88491373	0	2	2	intergenic	LOC389765(dist=33580),NAA35(dist=64664)	0.000117855	0.011785504
10	43120958	43121150	10_43121004	0	2	2	intronic	ZNF33B	0.000117855	0.011785504
10	99785634	99785826	10_99785782	2	0	2	intronic	CRTAC1	0.000117855	0.011785504
11	108006628	108006820	11_108006776	2	0	2	intronic	ACAT1	0.000117855	0.011785504
12	107659844	107660036	12_107659992	2	0	2	intergenic	CRY1(dist=172358),BTBD11(dist=52185)	0.000117855	0.011785504
13	25867023	25867215	13_25867069	0	2	2	intergenic	MTMR6(dist=5366),NUPL1(dist=8577)	0.000117855	0.011785504
13	61496413	61496605	13_61496561	2	0	2	intergenic	TDRD3(dist=348549),MIR3169(dist=277351)	0.000117855	0.011785504
15	51134767	51134959	15_51134813	0	2	2	intergenic	SPPL2A(dist=76904),AP4E1(dist=66036)	0.000117855	0.011785504
15	54830324	54830516	15_54830472	2	0	2	intronic	UNC13C	0.000117855	0.011785504
15	80596461	80596653	15_80596609	2	0	2	ncRNA_intronic	LOC283688	0.000117855	0.011785504
16	5695230	5695422	16_5695378	2	0	2	intergenic	FAM86A(dist=547590),RBFOX1(dist=373734)	0.000117855	0.011785504
16	22374840	22375032	16_22374988	1	1	2	intronic	CDR2	0.000117855	0.011785504
16	78310206	78310398	16_78310354	2	0	2	intronic	WWOX	0.000117855	0.011785504
17	16217120	16217312	17_16217268	2	0	2	intronic	PIGL	0.000117855	0.011785504
18	35302248	35302440	18_35302294	0	2	2	intergenic	MIR4318(dist=65117),LINC00669(dist=1484574)	0.000117855	0.011785504
18	52669031	52669223	18_52669077	0	2	2	intergenic	CCDC68(dist=42339),TCF4(dist=220465)	0.000117855	0.011785504
20	4539537	4539729	20_4539685	2	0	2	intergenic	ADRA1D(dist=310027),PRNP(dist=127092)	0.000117855	0.011785504
20	38224503	38224695	20_38224549	0	2	2	intergenic	LOC339568(dist=371159),MAFB(dist=1089948)	0.000117855	0.011785504
21	30555636	30555828	21_30555784	2	0	2	intergenic	MAP3K7CL(dist=7583),LINC00189(dist=10011)	0.000117855	0.011785504
22	19628114	19628306	22_19628262	1	1	2	intergenic	LOC150185(dist=73901),SEPT5(dist=73705)	0.000117855	0.011785504
22	28591457	28591649	22_28591605	2	0	2	intronic	TTC28	0.000117855	0.011785504
22	28609300	28609492	22_28609448	2	0	2	intronic	TTC28	0.000117855	0.011785504
X	16074714	16074906	X_16074760	0	2	2	intergenic	AP1S2(dist=201624),GRPR(dist=66644)	0.000117855	0.011785504
1	362708	362900	1_362856	1	0	1	intergenic	LOC100133331(dist=34276),OR4F29(dist=4783)	5.89275E-05	0.005892752
1	82067251	82067443	1_82067297	0	1	1	intergenic	NONE(dist=NONE),LPHN2(dist=198765)	5.89275E-05	0.005892752
1	103404413	103404605	1_103404561	1	0	1	intronic	COL11A1	5.89275E-05	0.005892752
1	104933142	104933334	1_104933290	1	0	1	intergenic	LOC100129138(dist=313598),NONE(dist=NONE)	5.89275E-05	0.005892752
1	114041886	114042078	1_114042034	1	0	1	intronic	MAGI3	5.89275E-05	0.005892752
1	145954393	145954585	1_145954439	0	1	1	intronic	NBPF10	5.89275E-05	0.005892752
1	147496112	147496303	1_147496158	0	1	1	intergenic	PDZK1P1(dist=11828),NBPF24(dist=78145)	5.89275E-05	0.005892752
1	201106043	201106235	1_201106089	0	1	1	intronic	TMEM9	5.89275E-05	0.005892752
1	225189518	225189710	1_225189666	1	0	1	intronic	DNAH14	5.89275E-05	0.005892752
1	247462130	247462320	1_247462276	1	0	1	intergenic	VN1R5(dist=41830),ZNF496(dist=1326)	5.89275E-05	0.005892752
2	34122019	34122211	2_34122065	0	1	1	intergenic	MYADML(dist=168782),NONE(dist=NONE)	5.89275E-05	0.005892752
2	35291340	35291532	2_35291488	1	0	1	intergenic	'ADML(dist=1338205),LOC100288911(dist=12903	5.89275E-05	0.005892752
2	156499550	156499717	2_156499673	1	0	1	intergenic	KCNJ3(dist=784810),NR4A2(dist=681251)	5.89275E-05	0.005892752
2	180771484	180771676	2_180771632	1	0	1	intergenic	ZNF385B(dist=45401),CWC22(dist=37952)	5.89275E-05	0.005892752
2	188082902	188083082	2_188082948	0	1	1	intergenic	ZSWIM2(dist=369052),CALCRL(dist=123722)	5.89275E-05	0.005892752
2	213300339	213300531	2_213300487	1	0	1	intronic	ERBB4	5.89275E-05	0.005892752
2	241345218	241345410	2_241345264	0	1	1	intergenic	OTOS(dist=265192),GPC1(dist=29831)	5.89275E-05	0.005892752
3	14027974	14028166	3_14028020	0	1	1	ncRNA_intronic	TPRXL	5.89275E-05	0.005892752
3	22085896	22086088	3_22086044	1	0	1	intergenic	ZNF385D(dist=293229),UBE2E2(dist=1158720)	5.89275E-05	0.005892752
3	38111082	38111274	3_38111230	1	0	1	intronic	DLEC1	5.89275E-05	0.005892752
3	101095489	101095681	3_101095535	0	1	1	intronic	SENP7	5.89275E-05	0.005892752
3	106696356	106696548	3_106696504	1	0	1	intergenic	CBLB(dist=1108618),LOC100302640(dist=132113	5.89275E-05	0.005892752
3	136950080	136950272	3_136950126	0	1	1	intergenic	IL20RB(dist=220201),SOX14(dist=532988)	5.89275E-05	0.005892752
3	148287430	148287622	3_148287578	1	0	1	intergenic	ZIC1(dist=1153073),AGTR1(dist=128060)	5.89275E-05	0.005892752
3	169402401	169402593	3_169402447	0	1	1	intergenic	MECOM(dist=20885),TERC(dist=79931)	5.89275E-05	0.005892752
4	16471010	16471202	4_16471056	0	1	1	intergenic	TAPT1-AS1(dist=211247),LDB2(dist=32089)	5.89275E-05	0.005892752
4	45554288	45554480	4_45554334	0	1	1	intergenic	GNPDA2(dist=825684),GABRG1(dist=483433)	5.89275E-05	0.005892752
4	69244932	69245124	4_69245080	1	0	1	intergenic	YTHDC1(dist=29257),TMPRSS11E(dist=68067)	5.89275E-05	0.005892752
4	112519625	112519817	4_112519671	0	1	1	intergenic	PITX2(dist=956393),C4orf32(dist=546862)	5.89275E-05	0.005892752
4	168164285	168164475	4_168164331	0	1	1	intergenic	SPOCK3(dist=8591),ANXA10(dist=849337)	5.89275E-05	0.005892752
5	17255964	17256141	5_17256010	0	1	1	intronic	BASP1	5.89275E-05	0.005892752
5	23934073	23934265	5_23934119	0	1	1	intergenic	PRDM9(dist=405414),CDH10(dist=553070)	5.89275E-05	0.005892752
5	54876291	54876483	5_54876439	1	0	1	intergenic	PPAP2A(dist=45567),SLC38A9(dist=45214)	5.89275E-05	0.005892752
5	69083620	69083812	5_69083768	1	0	1	intergenic	GUSBP3(dist=77497),SERF1A(dist=237284)	5.89275E-05	0.005892752
5	69242504	69242696	5_69242550	0	1	1	intergenic	GUSBP3(dist=236279),SERF1A(dist=78502)	5.89275E-05	0.005892752
5	69467109	69467301	5_69467257	1	0	1	ncRNA_intronic	SMA4	5.89275E-05	0.005892752
5	69663434	69663626	5_69663582	1	0	1	intergenic	SMA4(dist=77579),GTF2H2B(dist=47595)	5.89275E-05	0.005892752

5	69938092	69938284	5_69938138	0	1	1	intergenic	SMA5(dist=56590),SERF1A(dist=258332)	5.89275E-05	0.005892752		
5	70117564	70117756	5_70117610	0	1	1	intergenic	SMA5(dist=236062),SERF1A(dist=78860)	5.89275E-05	0.005892752		
5	72248598	72248790	5_72248746	1	0	1	intergenic	TNPO1(dist=38532),FCHO2(dist=3042)	5.89275E-05	0.005892752		
5	99026574	99026766	5_99026722	1	0	1	intergenic	100289230(dist=760010),LOC100133050(dist=688	5.89275E-05	0.005892752		
5	118742871	118743063	5_118742917	0	1	1	intergenic	TNFAIP8(dist=12624),HSD17B4(dist=45201)	5.89275E-05	0.005892752		
6	32937137	32937329	6_32937285	1	0	1	intronic	BRD2	5.89275E-05	0.005892752		
6	47890139	47890328	6_47890284	1	0	1	intronic	PTCHD4	5.89275E-05	0.005892752		
6	81948198	81948388	6_81948244	0	1	1	intergenic	BCKDHB(dist=892258),FAM46A(dist=507183)	5.89275E-05	0.005892752		
6	102249984	102250176	6_102250132	1	0	1	intronic	GRIK2	5.89275E-05	0.005892752		
6	113537263	113537455	6_113537411	1	0	1	intergenic	RFPL4B(dist=864914),MARCKS(dist=641096)	5.89275E-05	0.005892752		
6	151541947	151542139	6_151542095	1	0	1	intergenic	MTHFD1L(dist=119073),AKAP12(dist=19019)	5.89275E-05	0.005892752		
7	2545985	2546177	7_2546133	1	0	1	intergenic	CHST12(dist=71918),LFNG(dist=6010)	5.89275E-05	0.005892752		
7	36507941	36508133	7_36508089	1	0	1	intergenic	ANLN(dist=14690),AOAH(dist=44440)	5.89275E-05	0.005892752		
7	101122703	101122892	7_101122749	0	1	1	intronic	COL26A1	5.89275E-05	0.005892752		
8	121781	121973	8_121827	0	1	1	intergenic	OR4F21(dist=4804),RPL23AP53(dist=36498)	5.89275E-05	0.005892752		
8	92563902	92564094	8_92563948	0	1	1	intergenic	SLC26A7(dist=153571),RUNX1T1(dist=403227)	5.89275E-05	0.005892752		
9	27695643	27695835	9_27695689	0	1	1	intergenic	C9orf72(dist=121826),LINGO2(dist=252375)	5.89275E-05	0.005892752		
9	42413976	42414168	9_42414124	1	0	1	intergenic	ANKRD20A3(dist=2262),FAM95B1(dist=54445)	5.89275E-05	0.005892752		
10	10736746	10736938	10_10736792	0	1	1	intergenic	NONE(dist=NONE),SFTA1P(dist=89590)	5.89275E-05	0.005892752		
10	12136491	12136680	10_12136537	0	1	1	intronic	DHTKD1	5.89275E-05	0.005892752		
10	13473850	13474042	10_13473896	0	1	1	intergenic	SEPHS1(dist=83599),BEND7(dist=6568)	5.89275E-05	0.005892752		
10	104359065	104359257	10_104359111	0	1	1	intronic	SUFU	5.89275E-05	0.005892752		
10	106330497	106330689	10_106330645	1	0	1	intergenic	CCDC147(dist=115798),SORCS3(dist=70194)	5.89275E-05	0.005892752		
11	49748902	49749094	11_49749050	1	0	1	ncRNA_intronic	LOC440040	5.89275E-05	0.005892752		
11	129983153	129983345	11_129983199	0	1	1	intronic	APLP2	5.89275E-05	0.005892752		
12	16905572	16905764	12_16905720	1	0	1	intergenic	LMO3(dist=144573),SKP1P2(dist=235941)	5.89275E-05	0.005892752		
12	44502720	44502912	12_44502868	1	0	1	intronic	TMEM117	5.89275E-05	0.005892752		
12	67134253	67134445	12_67134299	0	1	1	intergenic	GRIP1(dist=61375),CAND1(dist=528742)	5.89275E-05	0.005892752		
12	120337763	120337913	12_120337869	1	0	1	intergenic	CIT(dist=22775),CCDC64(dist=89759)	5.89275E-05	0.005892752		
13	19855775	19855911	13_19855867	1	0	1	ncRNA_intronic	ANKRD26P3	5.89275E-05	0.005892752		
14	41655205	41655397	14_41655353	1	0	1	intergenic	FBXO33(dist=1753650),LRFN5(dist=421391)	5.89275E-05	0.005892752		
14	55553266	55553458	14_55553414	1	0	1	intergenic	MAPK1IP1L(dist=16503),LGALS3(dist=42501)	5.89275E-05	0.005892752		
14	103306548	103306740	14_103306696	1	0	1	intronic	TRAF3	5.89275E-05	0.005892752		
15	70798937	70799129	15_70798983	0	1	1	intergenic	TLE3(dist=408728),UACA(dist=147890)	5.89275E-05	0.005892752		
18	25822469	25822661	18_25822617	1	0	1	intergenic	CDH2(dist=65173),MIR302F(dist=2056239)	5.89275E-05	0.005892752		
18	28393063	28393255	18_28393109	0	1	1	intergenic	MIR302F(dist=514184),DSC3(dist=176923)	5.89275E-05	0.005892752		
18	30979192	30979384	18_30979238	0	1	1	intronic	CCDC178	5.89275E-05	0.005892752		
19	5124198	5124390	19_5124244	0	1	1	intronic	KDM4B	5.89275E-05	0.005892752		
21	21397016	21397208	21_21397062	0	1	1	intergenic	MPRSS15(dist=1621093),LINC00320(dist=71783	5.89275E-05	0.005892752		
22	50228568	50228708	22_50228614	0	1	1	intergenic	BRD1(dist=10163),ZBED4(dist=18863)	5.89275E-05	0.005892752		
X	19565380	19565572	X_19565528	1	0	1	intronic	SH3KBP1	5.89275E-05	0.005892752		
X	94547832	94548024	X_94547878	0	1	1	intergenic	AM133A(dist=1580606),LOC643486(dist=10441	5.89275E-05	0.005892752		
X	132959058	132959222	X_132959178	1	0	1	intronic	GPC3	5.89275E-05	0.005892752		
						16970						

Table S2. Vector type of each integration in Pt2

Gene	Vector type
SMARCC1	GCsapM-ADA
NAGA	LASN
LOC100129316	LASN
RGS9	LASN
DPP4	LASN

Table S3

GO Term

Immune system

GO:0051414, response to cortisol(Pathway)
GO:0002924, negative regulation of humoral immune response mediated by circulating immunoglobulin(Pathway)
GO:2000318, positive regulation of T-helper 17 type immune response(Pathway)
GO:0002829, negative regulation of type 2 immune response(Pathway)
GO:0002827, positive regulation of T-helper 1 type immune response(Pathway)
GO:0002890, negative regulation of immunoglobulin mediated immune response(Pathway)
GO:0002921, negative regulation of humoral immune response(Pathway) G
GO:0090713, immunological memory process(Pathway)
GO:0002923, regulation of humoral immune response mediated by circulating immunoglobulin(Pathway)
GO:0006957, complement activation, alternative pathway(Pathway)
GO:0051412, response to corticosterone(Pathway)
GO:2000316, regulation of T-helper 17 type immune response(Pathway)
GO:0002433, immune response-regulating cell surface receptor signaling pathway involved in phagocytosis(Pathway)
GO:0002825, regulation of T-helper 1 type immune response(Pathway)
GO:0002313, mature B cell differentiation involved in immune response(Pathway)
GO:0002507, tolerance induction(Pathway)
GO:0002230, positive regulation of defense response to virus by host(Pathway)
GO:0002828, regulation of type 2 immune response(Pathway)
GO:0002431, Fc receptor mediated stimulatory signaling pathway(Pathway)
GO:0048536, spleen development(Pathway)
GO:0042092, type 2 immune response(Pathway)
GO:0072538, T-helper 17 type immune response(Pathway)
GO:0042088, T-helper 1 type immune response(Pathway)
GO:0002920, regulation of humoral immune response(Pathway)
GO:0097720, calcineurin-mediated signaling(Pathway)
GO:0002823, negative regulation of adaptive immune response based on somatic recombination of immune receptors built from immunoglobulin superfamily domains(Pathway)
GO:0002820, negative regulation of adaptive immune response(Pathway)
GO:0002889, regulation of immunoglobulin mediated immune response(Pathway)
GO:0071384, cellular response to corticosteroid stimulus(Pathway)
GO:0002294, CD4-positive, alpha-beta T cell differentiation involved in immune response(Pathway)
GO:0002287, alpha-beta T cell activation involved in immune response(Pathway)
GO:0002293, alpha-beta T cell differentiation involved in immune response(Pathway)
GO:0002292, T cell differentiation involved in immune response(Pathway)
GO:0002312, B cell activation involved in immune response(Pathway)
GO:0001776, leukocyte homeostasis(Pathway)
GO:0032602, chemokine production(Pathway)
GO:0002824, positive regulation of adaptive immune response based on somatic recombination of immune receptors built from immunoglobulin superfamily domains(Pathway)
GO:0006958, complement activation, classical pathway(Pathway)
GO:0002698, negative regulation of immune effector process(Pathway)
GO:0002456, T cell mediated immunity(Pathway)
GO:0002821, positive regulation of adaptive immune response(Pathway)
GO:0002286, T cell activation involved in immune response(Pathway)
GO:0002455, humoral immune response mediated by circulating immunoglobulin(Pathway)
GO:0002224, toll-like receptor signaling pathway(Pathway)
GO:0006911, phagocytosis, engulfment(Pathway)
GO:0045089, positive regulation of innate immune response(Pathway)
GO:0006956, complement activation(Pathway)
GO:0031960, response to corticosteroid(Pathway)
GO:0002822, regulation of adaptive immune response based on somatic recombination of immune receptors built from immunoglobulin superfamily domains(Pathway)
GO:0002819, regulation of adaptive immune response(Pathway)
GO:0002285, lymphocyte activation involved in immune response(Pathway)
GO:0050777, negative regulation of immune response(Pathway)
GO:0016064, immunoglobulin mediated immune response(Pathway)
GO:0019724, B cell mediated immunity(Pathway)
GO:0045088, regulation of innate immune response(Pathway)
GO:0002699, positive regulation of immune effector process(Pathway)
GO:0002366, leukocyte activation involved in immune response(Pathway)
GO:0002263, cell activation involved in immune response(Pathway)
GO:0002429, immune response-activating cell surface receptor signaling pathway(Pathway)
GO:0002757, immune response-activating signal transduction(Pathway)
GO:0002768, immune response-regulating cell surface receptor signaling pathway(Pathway)
GO:0006959, humoral immune response(Pathway)
GO:0002697, regulation of immune effector process(Pathway)
GO:0002449, lymphocyte mediated immunity(Pathway)
GO:0002460, adaptive immune response based on somatic recombination of immune receptors built from immunoglobulin superfamily domains(Pathway)
GO:0002253, activation of immune response(Pathway)
GO:0002683, negative regulation of immune system process(Pathway)
GO:0002443, leukocyte mediated immunity(Pathway)
GO:0002764, immune response-regulating signaling pathway(Pathway)
GO:0050778, positive regulation of immune response(Pathway)
GO:0002252, immune effector process(Pathway)
GO:0002250, adaptive immune response(Pathway)
GO:0045087, innate immune response(Pathway)
GO:0050776, regulation of immune response(Pathway)
GO:0002684, positive regulation of immune system process(Pathway)
GO:0002520, immune system development(Pathway)
GO:0002682, regulation of immune system process(Pathway)
GO:0006955, immune response(Pathway)
GO:0002376, immune system process(Pathway)

Hematopoietic system

GO:1902035, positive regulation of hematopoietic stem cell proliferation(Pathway)
GO:1902033, regulation of hematopoietic stem cell proliferation(Pathway)
GO:0071425, hematopoietic stem cell proliferation(Pathway)
GO:0060218, hematopoietic stem cell differentiation(Pathway)
GO:1901532, regulation of hematopoietic progenitor cell differentiation(Pathway)
GO:0002244, hematopoietic progenitor cell differentiation(Pathway)
GO:0048534, hematopoietic or lymphoid organ development(Pathway)
GO:0002901, mature B cell apoptotic process(Pathway)
GO:0003159, morphogenesis of an endothelium(Pathway)
GO:0061154, endothelial tube morphogenesis(Pathway)
GO:0045446, endothelial cell differentiation(Pathway)
GO:0003158, endothelium development(Pathway)
GO:0030097, hemopoiesis(Pathway)

Supplemental figure

Figure S1

A

Pt1

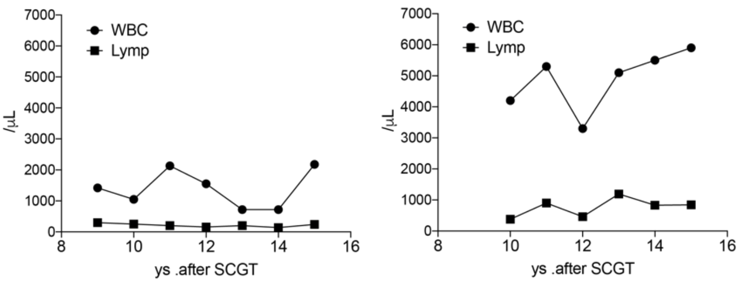

B

Pt2

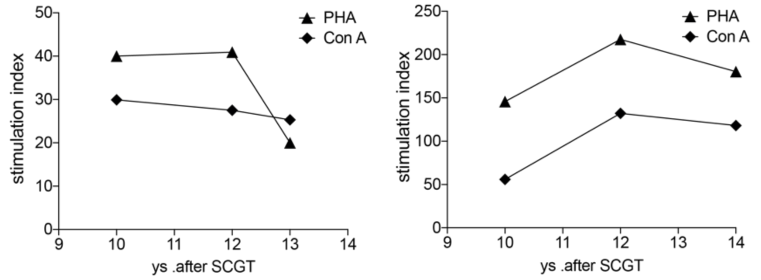

Figure S1. Levels of immune reconstitutions more than 10 years after SCGT in both patients. (A and B) Lymphocyte counts and the responses to mitogens were shown for both patients.

Figure S2

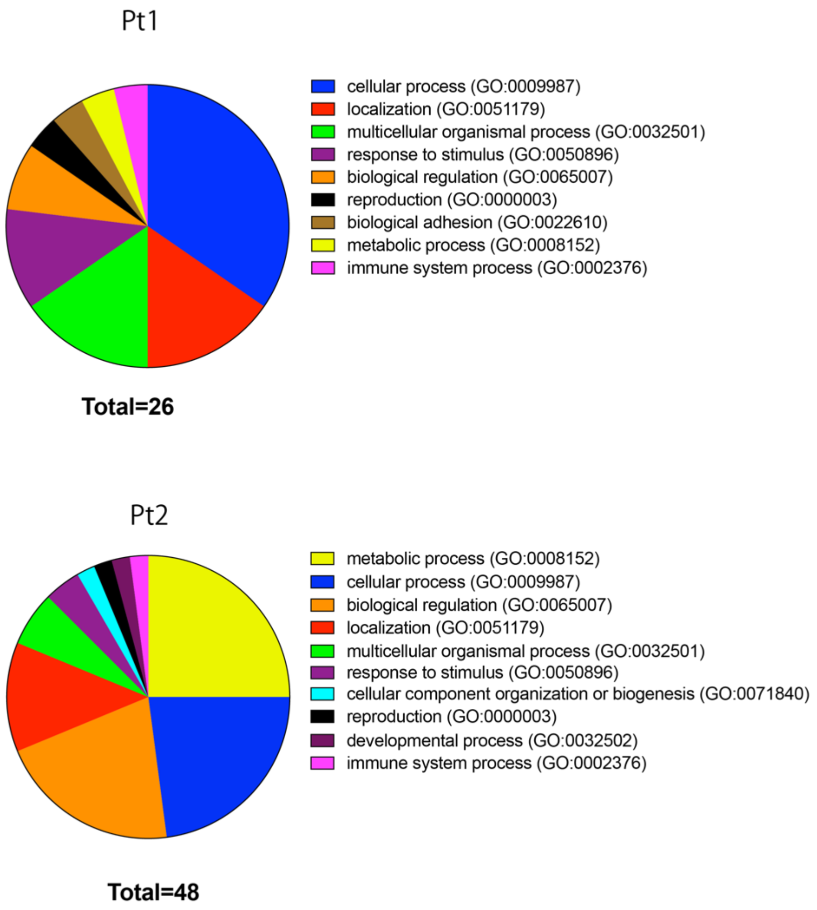

Figure S2. Biological categories of the genes proximal to the vector integration sites.

In gene ontology enrichment analysis, genes near ISs with total read numbers of more than 1% of all integrations were not statistically enriched in specific categories in both patients.

Figure S3

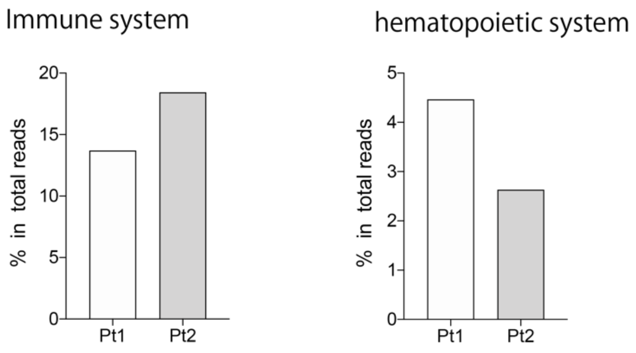

Figure S3. Function of genes hit by retroviral vector in the patients.

The functions of the genes with vector integration were analyzed. Frequencies of the genes that were involved in immune and hematopoietic system were shown.

Figure S4

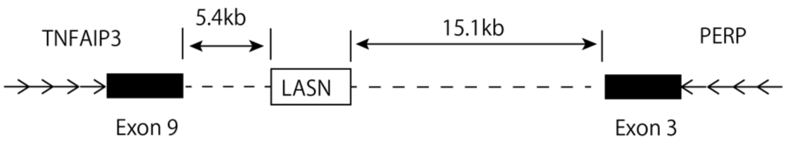

Figure S4. Structure of LASN integration between *TNFAIP3* and *PERP* in Pt2. LASN provirus located 5.4 kb downstream of *TNFAIP3* and 15.1 kb downstream of PERP, both of which have been reported as cancer genes.

Figure S5

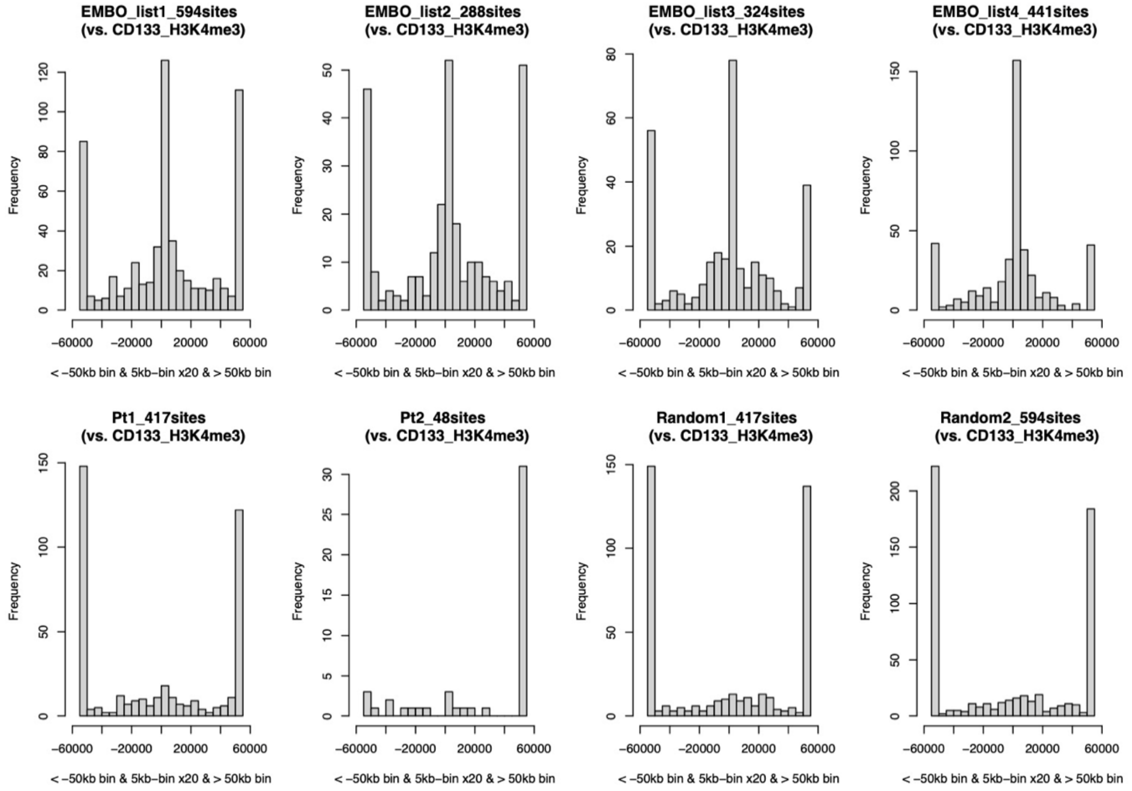

Figure S5. Distribution of the distance from the active transcriptional start site (TSS) in each integration. Each integration is analyzed for the distance from the sites of H3K4me3 modification corresponding to TSS of the genes. The data of the integration in the previous report (Biasco, et al[14]) and randomly selected region as controls were also shown.

Figure S6

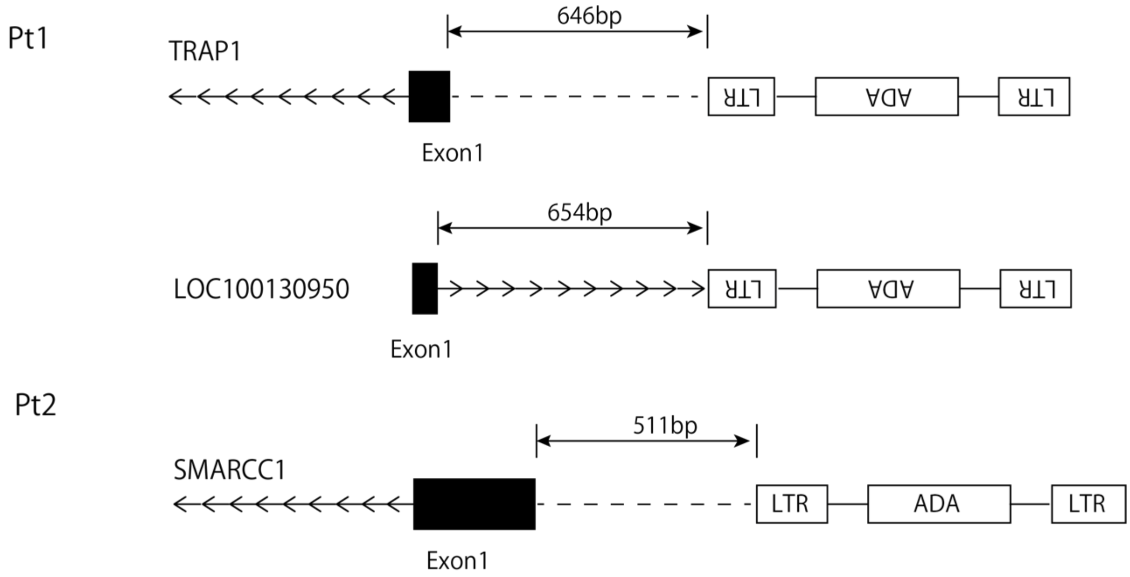

Figure S6. GCsapM-ADA provirus in the dominant clones in the patients. Integration of GCapM-ADA was detected in active transcriptional start site (TSS) of *TRAP1* and *LOC10012950* in Pt1, and *SMARCC1* in Pt2.

Supplemental file 1

LTR sequences of GCsapM-ADA and LASN

MSPV-LTR
sequence
as a part of GCsapADA sequence

>MSPV-LTR_444bp
AATGAAAGACCCCACCTGTAGGTTTGGCAAGCTAGCTTAAGTAACGCCATTTGCAAGGCATGGAAAAATACATAACTGAGAATAGAGAAGTTCAGATCAAGGTCAGG
AACAGAGAAACAGGAGAATATGGGCCAAACAGGATATCTGTGGTAAGCAGTTCCTGCCCCGCTCAGGGCCAAGAACAGTTGGAACAGGAGAATATGGGCCAAACAG
GATATCTGTGGTAAGCAGTTCCTGCCCCGCTCAGGGCCAAGAACAGATGGTCCCCAGATCGGTCCCGCCCTCAGCAGTTTCTAGAGAACCATCAGATGTTTCCAGG
GTGCCCCAAGGACCTGAAATGACCCTGTGCCTTATTTGAACTAACCAATCAGTTCGCTTCTCGCTTCTGTTCGCGCGCTTCTGCTCCCCGAGCTCAATAAAAGAGCC
CACAACCCCTCACTCGGC

MoMLV-LTR
sequence
as a part of the LASN sequence

>LASN-LTR (=MoMLV-_589bp)
TTTGAAAGACCCCACCCGTAGGTGGCAAGCTAGCTTAAGTAACGCCACTTTGCAAGGCATGGAAAAATACATAACTGAGAATAGGAAAGTTCAGATCAAGGTCAGGA
ACAAAGAAACAGCTGAATACCAAACAGGATATCTGTGGTAAGCGGTTCCTGCCCCGGCTCAGGGCCAAGAACAGATGAGACAGCTGAGTGATGGGCCAAACAGGAT
ATCGTGTGTAAGCAGTTCCTGCCCCGGCTCGGGGCCAAGAACAGATGGTCCCCAGATGCGGTCCAGCCCTCAGCAGTTTCTAGTGAATCATCAGATGTTTCCAGGG
TGCCCCAAGGACCTGAAAATGACCCTGTACCTTATTTGAACTAACCAATCAGTTCGCTTCTCGCTTCTGTTCGCGCGCTTCCGCTCTCCGAGCTCAATAAAAGAGCCC
ACAACCCCTCACTCGGCGCGCCAGTCTTCCGATAGACTGCGTCGCCCGGGTACCCGTATTCCCAATAAAGCCTCTTGCTGTTTGCATCCGAATCGTGGTCTCGCTG
TTCCTTGGGAGGGTCTCCTCTGAGTGATTGACTACCCACGACGGGGGTCTTTCATT

Custom SureSelect LTR baits for three LTR sequences (MoMLV-LTR, MPSV-LTR)
The baits also contains baits for SHH exons.
Design ID: 0812211

TargetID	ProbeID	Sequence	Replication
MoMLV-LTR	MoMLV-LTR_1-120	TTTGAAAGACCCCACCCGTAGGTGGCAAGCTAGCTTAAGTAACGCCACTTTGCAAGGCATGGAAAAATACATAACTGAGAATAGGAAAGTTCAGATCAAGGTCAGGAACAAAGAAACAGC	1
MoMLV-LTR	MoMLV-LTR_31-150	TAGCTTAAGTAACGCCACTTTGCAAGGCATGGAAAAATACATAACTGAGAATAGGAAAGTTCAGATCAAGGTCAGGAACAAAGAAACAGCTGAATACCAAACAGGATATCTGTGGTAAGC	1
MoMLV-LTR	MoMLV-LTR_61-180	GGAAAAATACATAACTGAGAATAGGAAAGTTCAGATCAAGGTCAGGAACAAAGAAACAGCTGAATACCAAACAGGATATCTGTGGTAAGCGGTTCCTGCCCCGGCTCAGGGCCAAGAACA	1
MoMLV-LTR	MoMLV-LTR_91-210	TCAGATCAAGGTCAGGAACAAAGAAACAGCTGAATACCAAACAGGATATCTGTGGTAAGCGGTTCCTGCCCCGGCTCAGGGCCAAGAACAGATGAGACAGCTGAGTGATGGGCCAAACAG	1
MoMLV-LTR	MoMLV-LTR_121-240	TGAATACCAAACAGGATATCTGTGGTAAGCGGTTCCTGCCCCGGCTCAGGGCCAAGAACAGATGAGACAGCTGAGTGATGGGCCAAACAGGATATCGTGTGTAAGCAGTTCCTGCCCCGG	1
MoMLV-LTR	MoMLV-LTR_151-270	GGTTCCTGCCCCGGCTCAGGGCCAAGAACAGATGAGACAGCTGAGTGATGGGCCAAACAGGATATCGTGTGTAAGCAGTTCCTGCCCCGGCTCGGGGCCAAGAACAGATGGTCCCCAGAT	1
MoMLV-LTR	MoMLV-LTR_181-300	GATGAGACAGCTGAGTGATGGGCCAAACAGGATATCGTGTGTAAGCAGTTCCTGCCCCGGCTCGGGGCCAAGAACAGATGGTCCCCAGATGCGGTCCAGCCCTCAGCAGTTTCTAGTGAA	1
MoMLV-LTR	MoMLV-LTR_211-330	GATATCGTGTGTAAGCAGTTCCTGCCCCGGCTCGGGGCCAAGAACAGATGGTCCCCAGATGCGGTCCAGCCCTCAGCAGTTTCTAGTGAATCATCAGATGTTTCCAGGGTGCCCCAAGGA	1
MoMLV-LTR	MoMLV-LTR_241-360	CTCGGGGCCAAGAACAGATGGTCCCCAGATGCGGTCCAGCCCTCAGCAGTTTCTAGTGAATCATCAGATGTTTCCAGGGTGCCCCAAGGACCTGAAAATGACCCTGTACCTTATTTGAAC	1
MoMLV-LTR	MoMLV-LTR_271-390	GCGGTCCAGCCCTCAGCAGTTTCTAGTGAATCATCAGATGTTTCCAGGGTGCCCCAAGGACCTGAAAATGACCCTGTACCTTATTTGAACTAACCAATCAGTTCGCTTCTCGCTTCTGTT	1
MoMLV-LTR	MoMLV-LTR_301-420	TCATCAGATGTTTCCAGGGTGCCCCAAGGACCTGAAAATGACCCTGTACCTTATTTGAACTAACCAATCAGTTCGCTTCTCGCTTCTGTTCGCGCGCTTCCGCTCTCCGAGCTCAATAAA	1
MoMLV-LTR	MoMLV-LTR_331-450	CCTGAAAATGACCCTGTACCTTATTTGAACTAACCAATCAGTTCGCTTCTCGCTTCTGTTCGCGCGCTTCCGCTCTCCGAGCTCAATAAAAGAGCCCACAACCCCTCACTCGGCGCGCCA	1
MoMLV-LTR	MoMLV-LTR_361-480	TAACCAATCAGTTCGCTTCTCGCTTCTGTTCGCGCGCTTCCGCTCTCCGAGCTCAATAAAAGAGCCCACAACCCCTCACTCGGCGCGCCAGTCTTCCGATAGACTGCGTCGCCCGGGTAC	1
MoMLV-LTR	MoMLV-LTR_391-510	CGCGCGCTTCCGCTCTCCGAGCTCAATAAAAGAGCCCACAACCCCTCACTCGGCGCGCCAGTCTTCCGATAGACTGCGTCGCCCGGGTACCCGTATTCCCAATAAAGCCTCTTGCTGTTT	1
MoMLV-LTR	MoMLV-LTR_421-540	AGAGCCCACAACCCCTCACTCGGCGCGCCAGTCTTCCGATAGACTGCGTCGCCCGGGTACCCGTATTCCCAATAAAGCCTCTTGCTGTTTGCATCCGAATCGTGGTCTCGCTGTTCCTTG	1
MoMLV-LTR	MoMLV-LTR_451-570	GTCTTCCGATAGACTGCGTCGCCCGGGTACCCGTATTCCCAATAAAGCCTCTTGCTGTTTGCATCCGAATCGTGGTCTCGCTGTTCCTTGGGAGGGTCTCCTCTGAGTGATTGACTACCC	1
MoMLV-LTR	MoMLV-LTR_470-589	CGCCCGGGTACCCGTATTCCCAATAAAGCCTCTTGCTGTTTGCATCCGAATCGTGGTCTCGCTGTTCCTTGGGAGGGTCTCCTCTGAGTGATTGACTACCCACGACGGGGGTCTTTCATT	1
MPSV-LTR	MPSV-LTR_1-120	AATGAAAGACCCCACCTGTAGGTTTGGCAAGCTAGCTTAAGTAACGCCATTTGCAAGGCATGGAAAAATACATAACTGAGAATAGAGAAGTTCAGATCAAGGTCAGGAACAGAGAAACAG	1
MPSV-LTR	MPSV-LTR_31-150	GCTAGCTTAAGTAACGCCATTTGCAAGGCATGGAAAAATACATAACTGAGAATAGAGAAGTTCAGATCAAGGTCAGGAACAGAGAAACAGGAGAATATGGGCCAAACAGGATATCTGTGG	1
MPSV-LTR	MPSV-LTR_61-180	TGGAAAAATACATAACTGAGAATAGAGAAGTTCAGATCAAGGTCAGGAACAGAGAAACAGGAGAATATGGGCCAAACAGGATATCTGTGGTAAGCAGTTCCTGCCCCGCTCAGGGCCAAG	1
MPSV-LTR	MPSV-LTR_91-210	TTCAGATCAAGGTCAGGAACAGAGAAACAGGAGAATATGGGCCAAACAGGATATCTGTGGTAAGCAGTTCCTGCCCCGCTCAGGGCCAAGAACAGTTGGAACAGGAGAATATGGGCCAAA	1
MPSV-LTR	MPSV-LTR_121-240	GAGAATATGGGCCAAACAGGATATCTGTGGTAAGCAGTTCCTGCCCCGCTCAGGGCCAAGAACAGTTGGAACAGGAGAATATGGGCCAAACAGGATATCTGTGGTAAGCAGTTCCTGCCC	1
MPSV-LTR	MPSV-LTR_151-270	TAAGCAGTTCCTGCCCCGCTCAGGGCCAAGAACAGTTGGAACAGGAGAATATGGGCCAAACAGGATATCTGTGGTAAGCAGTTCCTGCCCCGCTCAGGGCCAAGAACAGATGGTCCCCAG	1
MPSV-LTR	MPSV-LTR_181-300	AACAGTTGGAACAGGAGAATATGGGCCAAACAGGATATCTGTGGTAAGCAGTTCCTGCCCCGCTCAGGGCCAAGAACAGATGGTCCCCAGATCGGTCCCGCCCTCAGCAGTTTCTAGAGA	1
MPSV-LTR	MPSV-LTR_211-330	CAGGATATCTGTGGTAAGCAGTTCCTGCCCCGCTCAGGGCCAAGAACAGATGGTCCCCAGATCGGTCCCGCCCTCAGCAGTTTCTAGAGAACCATCAGATGTTTCCAGGGTGCCCCAAGG	1
MPSV-LTR	MPSV-LTR_241-360	CGCTCAGGGCCAAGAACAGATGGTCCCCAGATCGGTCCCGCCCTCAGCAGTTTCTAGAGAACCATCAGATGTTTCCAGGGTGCCCCAAGGACCTGAAATGACCCTGTGCCTTATTTGAAC	1
MPSV-LTR	MPSV-LTR_271-390	ATCGGTCCCGCCCTCAGCAGTTTCTAGAGAACCATCAGATGTTTCCAGGGTGCCCCAAGGACCTGAAATGACCCTGTGCCTTATTTGAACTAACCAATCAGTTCGCTTCTCGCTTCTGTT	1
MPSV-LTR	MPSV-LTR_301-420	ACCATCAGATGTTTCCAGGGTGCCCCAAGGACCTGAAATGACCCTGTGCCTTATTTGAACTAACCAATCAGTTCGCTTCTCGCTTCTGTTCGCGCGCTTCTGCTCCCCGAGCTCAATAAA	1
MPSV-LTR	MPSV-LTR_325-444	CCAAGGACCTGAAATGACCCTGTGCCTTATTTGAACTAACCAATCAGTTCGCTTCTCGCTTCTGTTCGCGCGCTTCTGCTCCCCGAGCTCAATAAAAGAGCCCACAACCCCTCACTCGGC	1

Baits for SHH

browser position chr7:155595548-155596430

track name="Target Regions" description="Agilent SureSelect DNA - SHH_151219 - Target regions of interest given as input to probe selection" color=0,128,0

chr7 155595547 155596430 SHH

chr7 155598979 155599261 SHH

chr7 155604506 155604977 SHH

track name="Missed" description="Agilent SureSelect DNA - SHH_151219 - Target regions of interest that will not be sequenced" color=128,0,0

chr7 155595568 155595608 SHH

chr7 155595728 155595781 SHH

chr7 155604923 155604950 SHH

track name="Covered" description="Agilent SureSelect DNA - SHH_151219 - Genomic regions expected to be sequenced" color=0,128,0

chr7 155595388 155595568 SHH

chr7 155595608 155595728 SHH

chr7 155595781 155596441 SHH

chr7 155598970 155599270 SHH

chr7 155604503 155604923 SHH

chr7 155604950 155605130 SHH

Supplemental file 2

Primers and probes for VCN analysis

Target	primer/probe	primer/probe ID	sequence
packaging signal ψ	Fw	Packaging Fw	GCAACCTATCTGTGTCTGTCC
	Rv	Packaging Rv	GGTCCGCCAGATACAGAG
	Probe	Packaging Probe	/56-FAM/TGCGGCCTGC/ZEN/GTCGTACTAGTTAG/3IABkFQ/
GCsapM-ADA	Fw	GCsapM-ADA Fw	TAGACGGCATCGCAGCTTGG
	Rv	GCsapM-ADA Rv	TCCGTCTAGGTGGACATGCAGT
	Probe	GCsapM-ADA Probe	/56-FAM/CGCCCGCCT/ZEN/TCGACAAGCCCAAAG/3YABkFQ/
RPP30	Fw	RPP30 Fw	TCCAGGAGGGAGAATTGATG
	Rv	RPP30 Rv	ATGGTCCGTCTCAGGAAATG
	Probe	RPP30 Probe	/HEX/TCCCTAGGT/ZEN/GGCCTGAGCAG/3IABkFQ/

Primers and probes for IS-specific ddPCR

Pt1

Gene name	primer/probe	primer/probe ID	sequence
TRAP1	Fw	ddTP Up Fw1	GAGGCACAGTCTCAAAAGGTC
	Rv	ddTP Up Rv1	TGAGTGATTGACTACCCACGA
	Probe	ddTP Up Probe 1	/56-FAM/GCTTGGGTTACAGCTTGCTT/3BHQ
LOC100130950	Fw	ddLOC Down Fw2	CAAACCTACAGGTGGGGTCT
	Rv	ddLOC Down Rv2	GCACTGACAGTTTTGCTTCG
	Probe	ddLOC Down Probe 2	/56-FAM/TAAGATGTCCAACCCCAAGC/3BHQ
ZFAND3	Fw	ddZFAND3 Fw	TGCATCCGAATCGTGGTCTC
	Rv	ddZFAND3 Rv	CCTGGCCAACAGTTGCTTTC
	Probe	ddZFAND3	/56-FAM/CCACGACGG/ZEN/GGGTCTTTCACCTTG/3IABkFQ/
DIP2A	Fw	ddDIP2A/USP4 Fw	TCCATGCCTTGCAAAATGGC
	Rv	ddDIP2A Rv	TCATTGAGGCTGGTCCAACC
	Probe	ddDIP2A/USP4	/56-FAM/GCTTGCCAA/ZEN/ACCTACAGGTGGGGT/3IABkFQ/
USP4	Fw	ddDIP2A/USP4 Fw	TCCATGCCTTGCAAAATGGC
	Rv	ddUSP4 Rv	TCTGGTCCCTTGAGTCTCCC
	Probe	ddDIP2A/USP4	/56-FAM/GCTTGCCAA/ZEN/ACCTACAGGTGGGGT/3IABkFQ/
LMO2	Fw	ddPCRLMO2Sense4	CTTGCAAAATGGCGTTA
	Rv	ddPCRLMO2Anti-sense4	GCTGGAATCGAGACAA
	Probe	Lower_Junc 2 LMO	/56-FAM/TTCACTTCCTTGCTAGAACTCAACA/3BHQ

Pt2

Gene name	primer/probe	primer/probe ID	sequence
SMARCC1	Fw	ddSMARCFW2	AATCGTGGTCTCGCTGTTCC
	Rv	ddSMARCRV2	TCGAAAGAGCCAGTGCAAGG
	Probe	SMARCC probe-new	/56-FAM/TTCAATTCCTGCCCCAGGG/3BHQ

Primers/probes for transcription levels of integrated genes

Patient	Gene name	**IDT Assay ID**
Pt1	TRAP1	Hs.PT.58.19693862
	GPX1	Hs.PT.58.39247474.g
	RAP1B	Hs.PT.58.26098638
Pt2	DPP4	Hs.PT.58.39108231.g
	TNFAIP3	Hs.PT.58.1824217
	PERP	Hs.PT.58.40332203
	MLLT10	Hs.PT.58.1482781
	SMARCC1	Hs.PT.58.3919159
Reference	TBP	Hs.PT.58v.39858774
